# Curvulones D–E and Bioactive Cytochalasans from *Macrozamia communis*-Associated Fungus *Penicillium* sp. MNP-GP-4

**DOI:** 10.3390/jof12100786

**Published:** 2026-10-20

**Authors:** Sherif S. Ebada, Mohamed S. Elnaggar, Ahmed M. Elissawy, Rémy Bertrand Teponno, Noureddine Bouras, Wael M. Afifi, Nehal Ibrahim, Abdel Nasser B. Singab, Nicole Teusch

**Affiliations:** 1 Institute of Pharmaceutical Biology and Biotechnology, Faculty of Mathematics and Natural Sciences, Heinrich Heine University Duesseldorf, 40225 Duesseldorf, Germany; mohamed.s.elnaggar@pharma.asu.edu.eg (M.S.E.); aelissawy@pharma.asu.edu.eg (A.M.E.); abdelnassersingab@pharma.asu.edu.eg (A.N.B.S.); 2 Department of Pharmacognosy, Faculty of Pharmacy, Ain Shams University, Cairo 11566, Egypt; nehal.sabry@pharma.asu.edu.eg; 3 Centre of Drug Discovery Research and Development, Ain Shams University, Cairo 11566, Egypt; 4 Department of Chemistry, Faculty of Science, University of Dschang, Dschang P.O. Box 67, Cameroon; remyteponno@gmail.com; 5 Laboratoire de Valorisation et Conservation des Ecosystèmes Arides (LVCEA), Faculté des Sciences de la Nature et de la Vie et Sciences de la Terre, Université de Ghardaia, Ghardaïa B.P. 455, Algeria; bouras.noureddine@univ-ghardaia.edu.dz; 6 Laboratoire de Biologie des Systèmes Microbiens (LBSM), Ecole Normale Supérieure de Kouba, Algiers 16308, Algeria; 7 Department of Pharmacognosy and Medicinal Plants, Faculty of Pharmacy, Al-Azhar University, Cairo 11884, Egypt; wael.afify@su.edu.eg; 8 Department of Pharmacognosy, Faculty of Pharmacy, Sinai University-Kantara Branch, Ismailia 41636, Egypt

**Keywords:** *Penicillium*, *Macrozamia communis*, curvularins, cytochalasan, cytotoxicity, PDAC, anti-tumor

## Abstract

*Macrozamia communis* (Australian cycad) is the most abundant cycad in Australia, yet its associated fungi lack chemical investigation. As a continuation of our ongoing studies on *M. communis*-associated fungi, an in-depth chemical exploration of the fermented fungal biomass of *Penicillium* sp. MNP-GP-4 afforded the identification of two previously undescribed curvularin derivatives, curvulones D (**1**) and E (**2**), together with three known metabolites, including curvularin (**3**) and two cytochalasan derivatives chaetoglobosins E and Fex (**4** and **5**). Chemical structures of the isolated compounds were elucidated based on comprehensive 1D and 2D NMR spectroscopic analyses and HR-(+)ESI-MS and comparison with the reported literature. All the isolated secondary metabolites were evaluated for their cytotoxic activity against a pancreatic duct adenocarcinoma (PDAC) cell line, where compounds **4** and **5** were the most active with IC_50_ values of 2.9 and 3.7 µM, respectively.

## 1. Introduction

Pancreatic ductal adenocarcinoma (PDAC), one of the most lethal malignancies today with a five-year survival rate of only 13%, is characterized by early metastasis, late diagnosis and intrinsic therapy resistance [1,2,3]. In addition to about half a million reported cases by 2020, more than 65,000 new PDAC cases were diagnosed in 2024 in the USA alone, and this number is expected to significantly increase in the upcoming years due to an aging population [4]. To date, cytotoxic chemotherapy with the combinatory regimen FOLFIRINOX (leucovorin, 5-fluorouracil, irinotecan and oxaliplatin) or, if intolerable, the combination of gemcitabine and capecitabine represents the first-line treatment for patients who are not eligible for surgical resection [5]. These drawbacks shed light on the intensive need for treatment alternatives against PDAC, including the search for novel secondary metabolites derived from endophytic fungi.

Curvularins comprise a distinct class of fungal polyketides featuring resorcyclic acid lactone (macrocylic lactone) with diverse oxidation, reduction, hydroxylation and/or halogenation patterns mainly on the macrocyclic lactone ring. According to the varying modifications, they can be grouped into distinct subclasses such as dehydrocurvularins, halogenated curvularins, hydroxylated curvularins, and curvularones (oxo-curvularins), among others [6,7]. Curvularins are widely isolated from different fungal genera derived from different sources such as sea sediments, marine invertebrates, medicinal plants, and agricultural crops with the major producing genera *Curvularia*, *Penicillium*, *Aspergillus*, *Alternaria* and *Fusarium* [8]. Previously, curvularins showed interesting biological activities including cytotoxic, anti-inflammatory and herbicidal effects [6,9].

On the other hand, cytochalasans represent another specific class of fungal secondary metabolites derived from mixed polyketide-non ribosomal peptide pathways (PKS-NRPS). The common structural core of cytochalasans is mainly derived from the polyketide pathway comprising tricyclic structure including one macrocyclic ring with 11,13 or 14 carbon atoms fused with isoindolone nucleus [10,11]. Cytochalasans are chemically classified into six chemical classes based on the specific amino acid residue linked to the tricyclic core, for example, cheatoglobosins with tryptophan residue (indole moiety), cytochalasins with phenyl alanine moiety, and alachalasins with alanine residue [12]. Cytochalasans were reported from different fungal species. They were first reported from *Helminthosporium dematioideum* in the late 1960s. Today, *Chaetomium*, *Aspergillus*, *Xylaria*, *Phomopsis* and *Penicillium* are common sources of cytochalasans [13]. Cytochalasans exhibit potential biological activities that are mainly centered on their inhibitory action on actin-polymerization, potentially affecting the cellular cytoskeleton. This is manifested in their potent cytotoxic, antimicrobial and anti-parasitic activities [13]. Interestingly, some cytochalasans, such as cytochalasin B, demonstrated an inhibitory action on glucose transporters, mainly GLUT1, making them a potential tool for studying metabolic processes as well as a useful pharmacophore in anti-diabetic drug development [14].

During our ongoing search for novel bioactive fungal metabolites, we came across a plant-associated strain of the genus *Penicillium*, whose preliminary screening revealed its content of curvularin and cytochalasin derivatives. Herein, we describe the isolation and characterization of five secondary metabolites, including two designated as previously undescribed, and their cytotoxicity against pancreatic cancer cells.

## 2. Materials and Methods

### 2.1. General Experimental Procedures

Optical rotation measurement was acquired using Perkin–Elmer-241 MC polarimeter (Perkin-Elmer, Shelton, CT, USA). Waters-G3-q-TOF was employed to record the HR-(+)ESI-MS spectra using a C18 Acquity UPLC BEH column (25 × 2.1 mm, 1.7 μm) (Waters, Milford, MA, USA). Medium-pressure liquid chromatography was performed using a Puriflash-4125 system equipped with a DAD detector using a prefilled normal-phase silica gel cartridge (Interchim, Montluçon Cedex, France). The final purification steps were performed using Shimadzu HPLC (Kyoto, Japan) preparative series combined with UV-PDA (photodiode array): SPD-M20A (column: 250 × 10 mm), Kromasil 100-10-C18 reversed-phase (RP-C18). The 1D and 2D nuclear magnetic resonance (NMR) spectra of isolated compounds were recorded on an Avance III 400 spectrometer (^1^H NMR: 400 MHz and ^13^C NMR: 100 MHz, Bruker, Billerica, MA, USA).

### 2.2. Fungal Material and Identification

The fungus *Penicillium* sp. MNP-GP-4 was isolated from the innermost tissues of the Australian strobile *Macrozamia communis* L.A.S. Johnson (Zamiaceae). A female strobile (40 × 20 cm) of *M. communis* was collected from Hunter Region Botanic Gardens (New South Wales, Australia) and was kindly authenticated by Mr. Ken Page (Chairman of the Hunter Region Botanic Gardens, NSW, Australia). For isolation of the associated fungal strain, according to the standard procedure, the fruit was thoroughly rinsed with distilled water, and then subjected to surface sterilization using 70% ethanol for 2 min. Samples from the innermost tissues were aseptically obtained as small pieces of the fruit through cutting using a sterilized blade before being pressed onto a malt agar plate (15 g/L malt extract, 15 g/L agar, 0.2 g/L chloramphenicol to suppress bacterial growth, pH adjusted to 7.4–7.8 using 10% NaOH). After incubation of agar plates at 25 °C, regular inspection revealed that the investigated fungal strain was observed to grow out of the fruit tissues. Pure fungal strains were grown by repeated re-inoculation on fresh culture media.

The fungal strain investigated was identified as *Penicillium* sp. MNP-GP-4 using a molecular biological protocol by DNA amplification and sequencing of the ITS region as previously described [15]. The obtained data of sequencing were submitted to GenBank with the accession number PZ358554.

### 2.3. Cultivation, Extraction and Isolation

Eight Erlenmeyer flasks (1 L), each containing 100 g rice and 110 mL water, were set aside overnight, then autoclaved for 20 min at 121 °C and used for fungal fermentation at 25 °C for six weeks under static conditions. For harvesting, each flask was soaked in EtOAc (Ethyl acetate) (3 × 500 mL), and the culture mass was mechanically cut into small pieces by a spatula. The flasks were set aside overnight and then shaken for 8 h at 150 rpm. The combined EtOAc extracts were then evaporated under vacuum at 40 °C to yield a brown oily residue. The obtained residue (3.2 g) was suspended in 90% aqueous methanol and partitioned with *n*-hexane to remove the fatty and oily material; the defatted 90% aqueous methanol layer was then evaporated under vacuum at 40 °C to yield 1.5 g of a reddish-brown solid residue. The defatted extract was then adsorbed on a minimal amount of normal-phase silica gel and applied to medium-pressure liquid chromatography, using normal-phase silica gel as a stationary phase and applying a stepwise gradient elution using *n*-hexane:DCM (dichloromethane) (100:0 to 0:100) over 15 min, followed by DCM:MeOH (100:0 to 95:5) over 15 min, DCM:MeOH (95:5 to 90:10) over 12 min, and DCM:MeOH (90:10 to 70:30) over 35 min to yield 80 fractions. The elution was monitored using a photodiode array (PDA) detector at a maximum absorption of 235–400 nm, the collected fractions were then monitored using thin-layer chromatography (TLC), and similar fractions were pooled together to yield four major fractions (F1–F4). Fraction F2 (120 mg) was further purified using semi-preparative reversed-phase HPLC, applying isocratic elution using 60% acetonitrile (MeCN) in deionized water as a mobile phase to yield **3** (15 mg, 0.46% yield, *t*_R_ = 10 min) and **4** (22 mg, 0.68% yield, *t*_R_ = 17 min). Similarly, fraction F3 (180 mg) was further purified with semi-preparative reversed-phase HPLC using the following gradient: 0–2 min 10% MeOH:Water; 2–5 min 25% MeOH:Water; 5–20 min 55% MeOH:Water; 20–30 min 65% MeOH:Water; 30–35 min 100% MeOH:Water. This yielded **1** (4 mg, 0.12% yield, *t*_R_ = 21 min), **2** (2.8 mg, 0.08% yield, *t*_R_ = 23.5 min), and **5** (4.1 mg, 0.12% yield, *t*_R_ = 27 min).

### 2.4. Spectral Data

#### 2.4.1. Curvulone D (**1**)

White amorphous solid; ${\left[\alpha \right]}_{D}^{20}$ −14.4 (*c* 0.1, MeOH); UV/Vis (MeOH): λ_max_ 206, 237, 288 nm; NMR data (^1^H: 400 MHz, ^13^C: 100 MHz, acetone-*d*_6_) (see Table 1); HR-(+)ESI-MS: *m*/*z* 329.0986 [M + Na]^+^ (cacld. 329.0996 for C_16_H_18_NaO_6_^+^; mass error −2.92 ppm).

#### 2.4.2. Curvulone E (**2**)

White amorphous solid; ${\left[\alpha \right]}_{D}^{20}$ −26.9 (*c* 0.1, MeOH); UV/Vis (MeOH): λ_max_ 218, 271, 301 nm, 203 nm; NMR data (^1^H: 400 MHz, ^13^C: 100 MHz, DMSO-*d*_6_); HR-(+)ESI-MS: *m*/*z* 347.14517 [M + Na]^+^ (cacld. 347.14651 for C_17_H_24_NaO_6_^+^ mass error −3.86 ppm).

#### 2.4.3. (15*S*)-Curvularin (**3**)

White solid powder; ${\left[\alpha \right]}_{D}^{20}$ −17.4 (*c* 0.1, MeOH); UV/Vis (MeOH): λ_max_ 222, 273, 303 nm; NMR data (^1^H: 400 MHz, ^13^C: 100 MHz, DMSO-*d*_6_) comparable to the previously described spectral data [16,17]; LR-(+)ESI-MS: *m*/*z* 293.04 [M + H]^+^.

#### 2.4.4. Chaetoglobosin E (**4**)

White solid powder; UV/Vis (MeOH): λ_max_ 220, 282 nm; NMR data (^1^H: 400 MHz, ^13^C: 100 MHz, DMSO-*d*_6_) comparable to the previously described spectral data [18,19]; LR-(+)ESI-MS: *m*/*z* 531.26 [M+H]^+^.

#### 2.4.5. Chaetoglobosin Fex (**5**)

White solid powder; UV/Vis (MeOH): λ_max_ 222, 282 nm; NMR data (^1^H: 400 MHz, ^13^C: 100 MHz, chloroform-*d*) comparable to the previously described spectral data [20]; LR-(+)ESI-MS: *m*/*z* 531.26 [M+H]^+^.

### 2.5. Cell Culture and Biological Assays

The PANC-1 cell line, acquired from the American Type Culture Collection (ATCC), was cultured in Dulbecco’s modified Eagle’s medium (DMEM) (#41965039, Gibco Grand Island, NY, USA) supplemented with 10% fetal bovine serum (FBS) (#10270-106, Gibco, Schwerte, Germany), as well as 1% penicillin–streptomycin (pen-strep) (#15140122, Gibco, Schwerte, Germany) as described in our previous studies [21,22]. Cell viability in PANC-1 cells was assessed using the PrestoBlue HS Cell Viability Assay (#P50201, Thermo Scientific, Waltham, MA, USA) in 96-well plates (#655090, Greiner Bio-One, Kremsmünster, Austria), seeding 5000 cells of PANC-1 cells per well as previously reported [23]. Compounds were incubated for 72 h. Fluorescence values were recorded using the Tecan SPARK instrument (Tecan Group, Männedorf, Switzerland). All experiments were conducted in at least three independent experiments. Dose–response curves were generated, and IC_50_ values were determined by nonlinear regression analysis using GraphPad Prism 8.0.2 software (GraphPad Software, Boston, MA, USA).

## 3. Results

### 3.1. Isolation and Structure Elucidation of Secondary Metabolites

**Figure 1:**
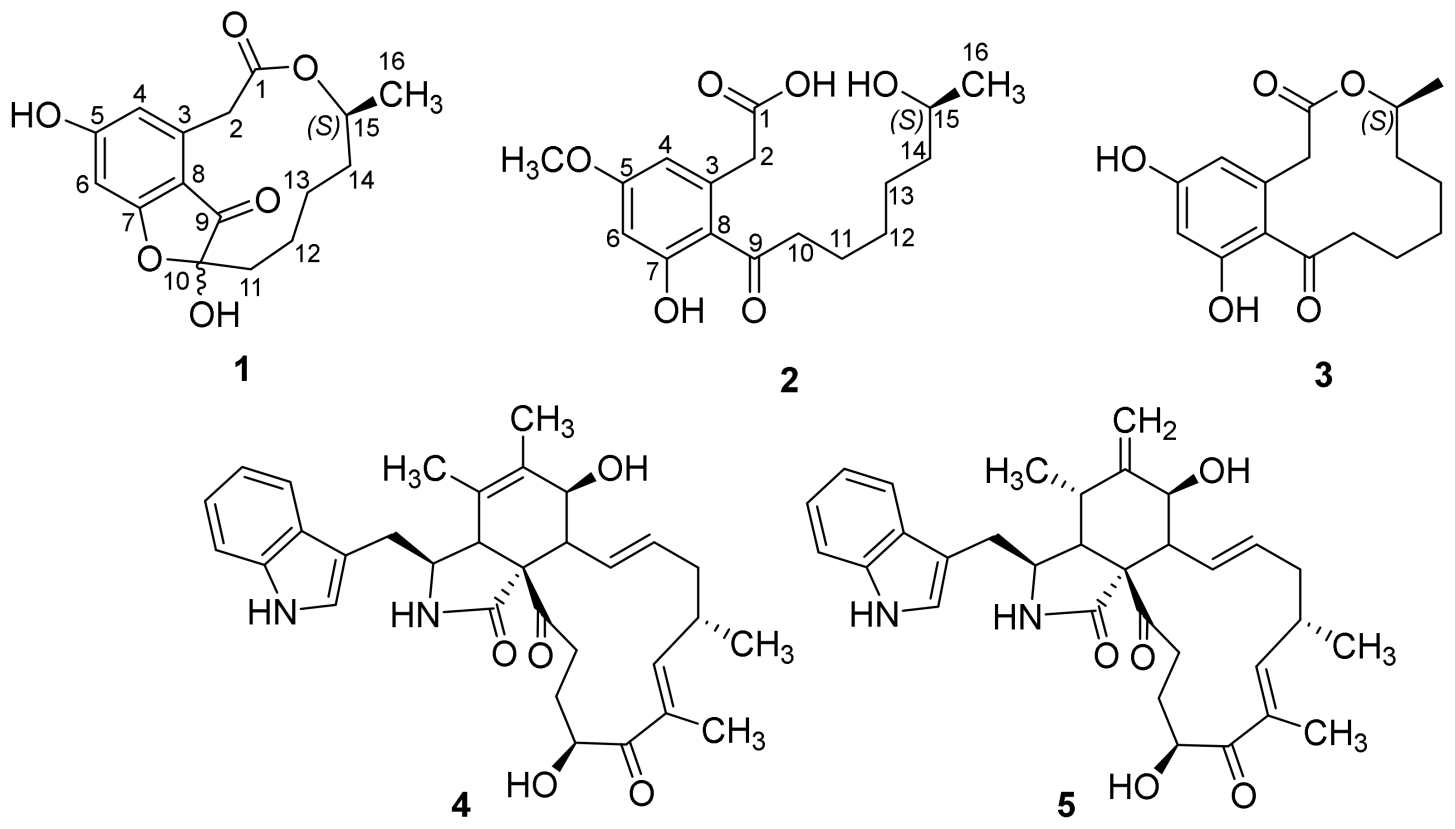
Chemical structures of **1**–**5**.

Chemical investigation of *Penicillium* sp. MNP-GP-4 led to the isolation and structure elucidation of two previously unreported curvularin derivatives (**1** and **2**) and three known compounds identified as curvularin (**3**) [16,17], as well as chaetoglobosins E (**4**) [18,19] and Fex (**5**) [20] (Figure 1).

Compound **1** was obtained as a white amorphous solid. Its molecular formula was deduced to be C_16_H_18_O_6_ through its HR-(+)ESI-MS, which displayed a sodium adduct peak [M + Na]^+^ at *m*/*z* 329.0986 (calculated 329.0996), indicating eight degrees of unsaturation. The ^1^H NMR spectral data of **1** (Table 1, Figure S2) showed, in addition to two aromatic proton signals at δ_H_ 6.37 (d, *J* = 1.9 Hz, H-6) and 6.45 (d, *J* = 1.9 Hz, H-4), a doublet methyl group at δ_H_ 1.01 (d, *J* = 6.2 Hz, CH_3_-16), an oxymethine proton at δ_H_ 4.74 (ddd, *J* = 9.5, 6.3, 3.1 Hz, H-15), and two diastereotopic proton signals at δ_H_ 3.55 (d, *J* = 17.1 Hz, Hα-2) and 4.13 (d, *J* = 17.1 Hz, Hβ-2). Some resonances observed between δ_H_ 2.14 and 0.90 ppm were ascribed to methylene moieties (Table 1). The ^13^C NMR spectrum exhibited signals of six quaternary carbon resonances, including a ketoncarbonyl (δ_C_ 198.2, C-9), a carboxycarbonyl (δ_C_ 169.1, C-1) and four aromatic (δ_C_ 173.8 (C-7), 168.0 (C-5), 137.8 (C-3), and 113.5 (C-8)). The ^13^C NMR spectral data of **1** (Table 1) also revealed two olefinic methines at δ_C_ 113.2 (C-4) and 97.0 (C-6), as well as a hemiketal carbon at δ_C_ 107.9 (C-10) [24,25].

**Figure 2:**
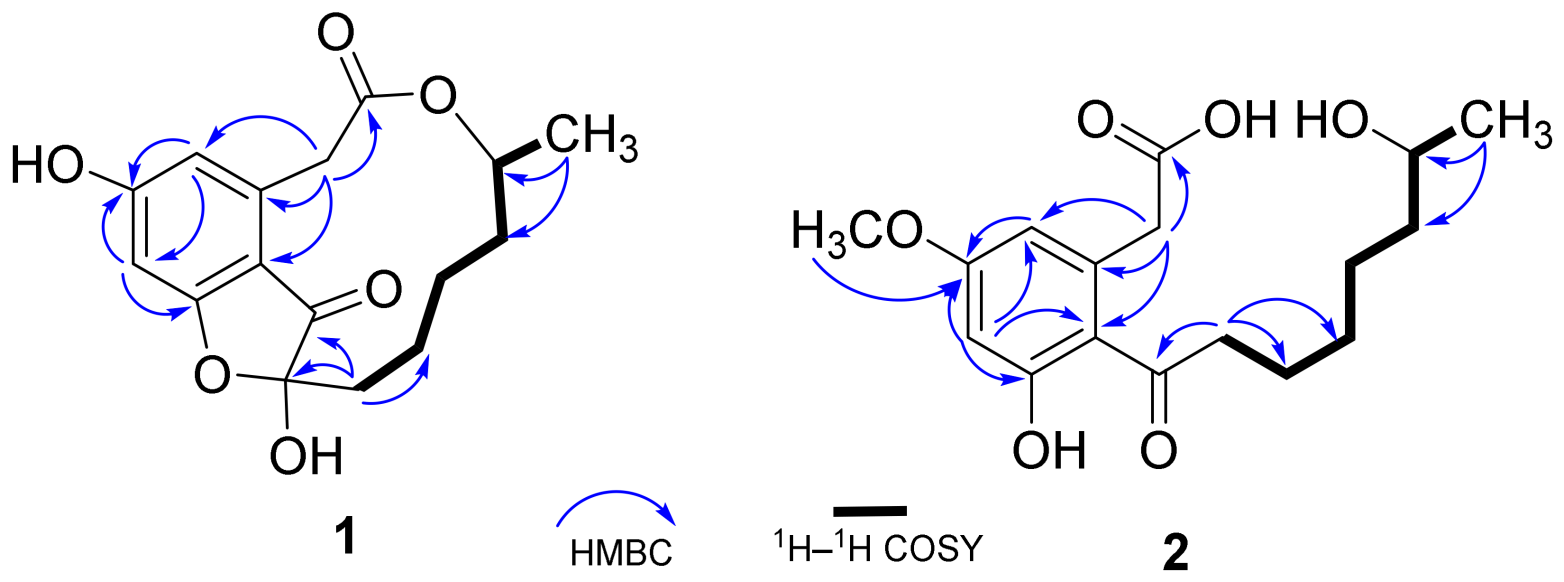
Selected HMBC and ^1^H–^1^H COSY correlations for **1** and **2**.

The remaining carbon resonances were attributed to an oxygenated *sp*^3^ methine carbon at δ_C_ 72.6 (C-15), five methylenes at δ_C_ 39.0 (C-2), 35.8 (C-11), 34.3 (C-14), 24.1 (C-13), and 23.6 (C-12), and a methyl group at δ_C_ 20.0 (C-16). A detailed analysis of the ^1^H and ^13^C NMR, ^1^H–^1^H COSY, HSQC, and HMBC spectra revealed that compound **1** was related to curvulone A, a curvularin-type macrolide previously isolated from the marine-derived fungus *Curvularia* sp. [26]. The main difference between **1** and curvulone A was found at C-10 and C-12. A hydroxyl group was linked to C-10 in compound **1**, while a carbonyl group was present at C-12 of curvulone A [26]. In the HMBC spectrum (Figure 2), key correlations were detected from proton signals at δ_H_ 3.55 (d, *J* = 17.1 Hz, Hα-2) and 4.13 (d, *J* = 17.1 Hz, Hβ-2) to carbon peaks at δ_C_ 169.1 (C-1), 137.8 (C-3), 113.2 (C-4), and 113.5 (C-8); from a proton signal at δ_H_ 6.37 (d, *J* = 1.9 Hz, H-6) to carbon signals at δ_C_ 173.8 (C-7), 168.0 (C-5), 113.5 (C-8), and 113.2 (C-4); and from a methyl group at δ_H_ 1.01 (d, *J* = 6.2 Hz, CH_3_-16) to carbon atoms at δ_C_ 72.6 (C-15) and 34.3 (C-14). Although the HMBC correlation from the proton at δ_H_ 4.74 (ddd, *J* = 9.5, 6.3, 3.1 Hz, H-15) to the carbon at δ_C_ 169.1 (C-1) was not depicted, the deshielded chemical shifts observed for this proton and carbon C-15 (δ_C_ 72.6) supported the argument that it was implicated in the lactonization.

The absolute configuration of curvularin (**3**) was determined as (15*S*), based on comparing its measured optical rotation value with the reported literature [16]. Being isolated from the same source and presumably through the same biosynthetic pathway, the absolute configuration of **1** was tentatively determined as (15S*). This can be confirmed by the optical rotation measurement of **1** with ${\left[\alpha \right]}_{D}^{20}$ −14.4, comparable to the measured optical rotation of **3** (${\left[\alpha \right]}_{D}^{20}$ −17.4). Based on the above data, compound **1** was elucidated as a previously unreported curvularin-type macrolide, to which the trivial name curvulone D was attributed.

The molecular formula of compound **2** (isolated as a white amorphous solid) was determined as C_17_H_24_O_6_ from the HR-(+)ESI-MS analysis, which showed the sodium adduct ion at *m*/*z* 347.14517 [M + Na]^+^ (calculated 347.14651), indicating six degrees of unsaturation. Its ^1^H NMR and the ^1^H–^1^H COSY spectra (Figure 2) exhibited resonances for two meta-coupled aromatic protons at δ_H_ 6.30 (d, *J* = 2.2 Hz, H-4) and 6.37 (d, *J* = 2.2 Hz, H-6), suggesting the presence of a 1,2,3,5-tetrasubstituted benzene ring in the molecule. This spectrum also displayed signals for an oxygenated methyl group at δ_H_ 3.70 (3H, s, 5-OMe), a methylene singlet at δ_H_ 3.45 (2H, s, H_2_-2), a methyl doublet at δ_H_ 1.02 (d, *J* = 6.2, H_3_-16), as well as an oxymethine proton at δ_H_ 3.57 (m, H-15). The remaining signals were attributed to five aliphatic methylenes at δ_H_ 2.80 (t, *J* = 7.4 Hz, H_2_-10), 1.52 (m, H_2_-11), 1.22 (m, H_2_-12), 1.37 (m, H_2_-13), and 1.30 (m, H_2_-14). The ^13^C NMR spectrum of **2** was closely comparable to that of **1**, with resonances of six aromatic carbon atoms observed at δ_C_ 136.5 (C-3), 108.8 (C-4), 160.9 (C-5), 100.2 (C-6), 157.4 (C-7), and 122.4 (C-8); a ketocarbonyl at δ_C_ 206.5 (C-9); a carboxyl group at δ_C_ 172.9 (C-1); a methyl carbon at δ_C_ 24.1 (C-16); and a hydroxymethine at δ_C_ 66.2 (C-15). It has also revealed the presence of a methoxy carbon at δ_C_ 55.5 (5-OMe) and six aliphatic methylene carbon atoms at δ_C_ 39.8 (C-2), 43.9 (C-10), 24.2 (C-11), 29.4 (C-12), 25.8 (C-13), and 39.5 (C-14) (Table 1).

These data exposed that compound **2** is a hydroxycarboxylic acid derivative of curvularin containing a methoxy group. The full assignment of ^1^H and ^13^C chemical shifts was achieved by the comprehensive interpretation of 1D (^1^H/^13^C) and 2D (^1^H–^1^H COSY, HSQC, and HMBC) NMR spectra. Some key HMBC correlations (Figure 2) were observed from H_2_-2 (δ_H_ 3.45, s) to carbon resonances at δ_C_ 172.9 (C-1), 136.5 (C-3), 108.8 (C-4), and 122.4 (C-8); from a proton signal at δ_H_ 6.37 (d, *J* = 2.2, H-6) to carbon signals at δ_C_ 160.9 (C-5), 157.4 (C-7), 108.8 (C-4), and 122.4 (C-8); from the methylene proton resonances at δ_H_ 2.80 (t, *J* = 7.4, H_2_-10) to C-9 (δ_C_ 206.5), C-11 (δ_C_ 24.2) and C-12 (δ_C_ 29.4); and from the methyl group at δ_H_ 1.02 (d, *J* = 6.2, H_3_-16) to carbon signals at δ_C_ 66.2 (C-15) and 39.5 (C-14). The location of the methoxy group was evidenced by the HMBC correlation from the proton signals at δ_H_ 3.70 (s, 5-OMe) to carbon C-5 (δ_C_ 160.9).

According to the measured optical rotation value of **2** (${\left[\alpha \right]}_{D}^{20}$ −26.9), it was cohering to that measured for (15*S*)-curvularin (**3**) as well as its reported value in the literature [27]. The absolute configuration at C-15 in **2** was tentatively identified as (15S*). Based on the obtained results, compound **2** was elucidated as a previously undescribed acyclic curvularin-type derivative trivially named curvulone E.

### 3.2. Biological Activity of Secondary Metabolites

**Figure 3:**
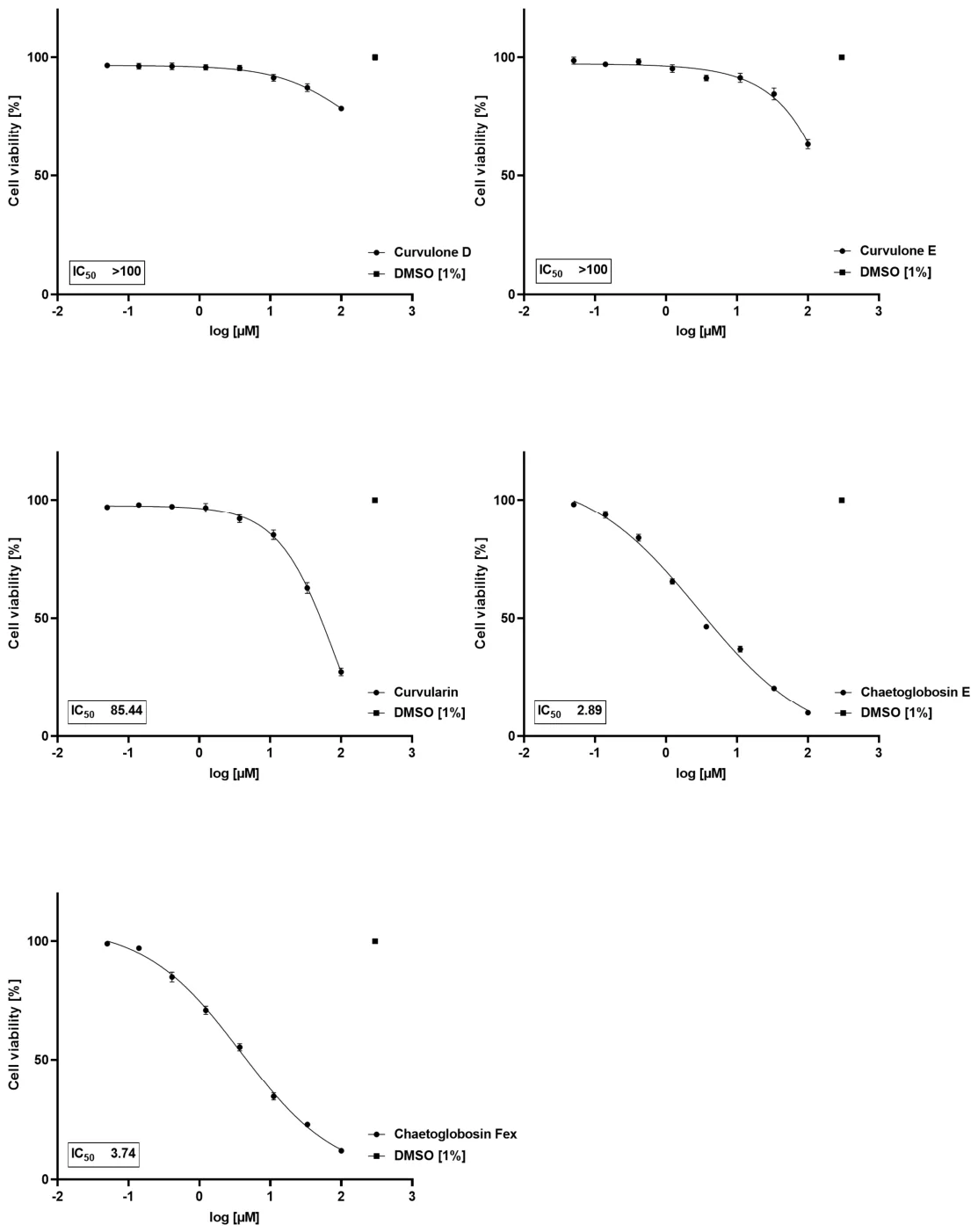
Viability was assessed in the PDAC cell line PANC-1 after 72 h of treatment. IC_50_ values were determined by nonlinear regression using GraphPad Prism. Data are presented as the percentage of viable cells relative to the solvent control DMSO. Error bars indicate the standard errors of the mean of at least three independent biological repeats.

All isolated compounds were assessed for their cytotoxic activity against the pancreatic duct adenocarcinoma (PDAC) cell line PANC-1. The obtained results (Figure 3 and Table 2) revealed that significant activity was noticed for chaetoglobosins E (**4**) and Fex (**5**) with IC_50_ values of 2.9 and 3.7 µM, respectively, whereas curvularin (**3**) revealed only moderate efficacy with an IC_50_ value of 85.4 µM.

## 4. Discussion

*M. communis* proved to be a prolific source of fungal strains with an interesting pool of secondary metabolites. In a recent study, we reported previously undescribed antimicrobial and cytotoxic mycophenolic acid derivatives from *Penicillium* sp. associated with *M. communis* [28]. In the present report, a different *M. communis*-associated *Penicillium* sp. was chemically and biologically investigated, revealing two distinct classes of secondary metabolites, mainly cytochalasan alkaloids and curvularin derivatives. Among the elucidated curvularin derivatives in this study, curvulones D and E were presented as previously undescribed natural products. All the isolated compounds were investigated for their cytotoxic potential against pancreatic cancer cell line PANC-1 as a part of our ongoing initiative for the discovery of lead drug candidates against pancreatic duct adenocarcinoma (PDAC). As depicted in the cytotoxicity assay results, only **3**, **4** and **5** revealed significant activity, with cytochalasan derivatives (**4**/**5**) being the most potent.

Previous reports showed that curvularin derivatives revealed variable cytotoxic activities as being sensitive to minor changes in their chemical structures, either in the aromatic ring or the macrocyclic scaffold. For example, the C-10/C-11 unsaturation in dehydrocurvularin significantly enhanced cytotoxicity compared to the saturated curvularin. Another example is the hydroxylation in 11-hydroxycurvularin, affording potent cytotoxicity compared to curvularin [9,17]. In the present report, the cytotoxicity of the identified curvularins (**1**–**3**) were significantly low compared to the reported literature, where only curvulone (**3**) revealed moderate cytotoxicity with 85.4 μM. The main structural variations in **1** compared to **3** is the presence of hemiacetal at C-10, bridging the aromatic ring at 7-OH with C-10 and creating a cyclofuranone ring; this variation is the key reason for the different cytotoxic features among **1**–**3**.

On the other hand, the cytochalasin derivatives, chaetoglobosins E (**4**) and Fex (**5**), were reported to have potent cytotoxicity against different cancer cell lines, yet the potency may vary according to the assessed cell line [29,30,31]. In the present study, both revealed potent activity against the PANC-1 cell line which is, to the best of our knowledge, the first report for their cytotoxic evaluation against pancreatic cancer. In 2022, Zhang, Chen and their fellow team members studied the cytotoxic mechanisms of several cytochalasans to reveal multiple mechanisms, with apoptosis induction being the most common mechanism and disruption of actin microfilament dynamics, causing cell cycle arrest at the G_2_/M phase [29]. Worth noting is that, in subsequent studies, cytotoxicity against non-tumor cells need to be addressed to determine tumor cell specificity.

## Figures and Tables

**Table 1:** ^1^H and ^13^C NMR data of **1** and **2** (*δ* in ppm, *J* in Hz).

Position	1 *		2 *	
	δ_C_, ^a,c^ Type	δ_H_, ^b^ (Multiplicity, *J* in Hz)	δ_C_, ^a,c^ Type	δ_H_, ^b^ (Multiplicity, *J* in Hz)
1	169.1, CO	/	172.9, CO	/
2	39.0, CH_2_	α 3.55 (d, 17.1) β 4.13 (d, 17.1)	39.8, CH_2_	3.45 (brs)
3	137.8, C	/	136.5, C	/
4	113.2, CH	6.45 (d, 1.9)	108.8, CH	6.30 (d, 2.2)
5	168.0, C	/	160.9, C	/
6	97.0, CH	6.37 (d, 1.9)	100.2, CH	6.37 (d, 2.2)
7	173.8, C	/	157.4, C	/
8	113.5, C	/	122.4, C	/
9	198.2, CO	/	206.5, CO	/
10	107.9, C	/	43.9, CH_2_	2.80 (t, 7.4)
11	35.8, CH_2_	α 1.82 (m); β 2.14 (m)	24.2, CH_2_	1.52 (m)
12	23.6, CH_2_	α 0.96 (m); β 1.46 (m)	29.4, CH_2_	1.22 (m)
13	24.1, CH_2_	α 1.03 (m); β 1.28 (m)	25.8, CH_2_	1.37 (m)
14	34.3, CH_2_	α 0.90 (m); β 1.63 (m)	39.5, CH_2_	1.30 (m)
15	72.6, CH	4.74 (ddd, 9.5, 6.3, 3.1)	66.2, CH	3.57 (m)
16	20.0, CH_3_	1.01 (d, 6.2)	24.1, CH_3_	1.02 (d, 6.2)
5-OCH_3_			55.5, CH_3_	3.70 (s)

Measured at ^a^ 100 MHz for ^13^C and ^b^ 400 MHz for ^1^H. ^c^ Assignment confirmed by HSQC and HMBC. * Compound **1** measured in acetone-*d*_6_; compound **2** measured in DMSO-*d*_6_.

**Table 2:** Cytotoxic activity (IC_50_ in µM) against the PDAC cell line PANC-1 of **1**–**5**.

Compound	Trivial Name	Chemical Class	IC_50_ [µM]
**1**	Curvulone D	Curvularin-type macrolide	>100
**2**	Curvulone E	Curvularin-type acyclic polyketide	>100
**3**	Curvularin	Curvularin-type macrolide	85.4
**4**	Chaetoglobosin E	Indolo-cytochalasan alkaloid	2.9
**5**	Chaetoglobosin Fex	Indolo-cytochalasan alkaloid	3.7
Positive control	Gemcitabine		0.04

## Data Availability

The DNA sequences are deposited in GenBank (https://www.ncbi.nlm.nih.gov/genbank/ (accessed on 1 May 2026)), and all other relevant data are included in the Supplementary Information.

## Supplementary Materials

The following supporting information can be downloaded at: https://www.mdpi.com/article/10.3390/jof12100786/s1, Figures S1–S33: LR-/HR-ESI-MS and 1D/2D NMR spectra of **1**–**5** in methanol-*d*_4_ or chloroform-*d* at 400 (for ^1^H) and 100 (for ^13^C) MHz; Figure S34: ITS sequence of the fungal strain MNP-GP-4.
