# Supplementary material for: Curvulones D–E and Bioactive Cytochalasans from Macrozamia communis-Associated Fungus Penicillium sp. MNP-GP-4
Source: J Fungi (Basel). 2026 Oct 20;12(10):786. doi: 10.3390/jof12100786 (PMC13705659; doi:10.3390/jof12100786)
Supplement: Supplementary file 1 [file jof-12-00786-s001.zip › jof-4574680-supplementary.pdf]

Supplementary Materials for:

Curvulones D–E and bioactive cytochalasans from *Macrozamia communis*-associated fungus *Penicillium* sp. MNP-GP-4

Sherif S. Ebada [1,2,#*], Mohamed S. Elnaggar [1,2,#], Ahmed M. Elissawy [1,2,3,#], , Rémy Bertrand Teponno [4], Noureddine Bouras [5,6], Wael M. Afifi [7,8], Nehal Ibrahim[2], Abdel Nasser B. Singab [1,2,3] and Nicole Teusch [1*]

[1] Institute of Pharmaceutical Biology and Biotechnology, Faculty of Mathematics and Natural Sciences, Heinrich Heine University Duesseldorf, 40225 Duesseldorf, Germany; nicole.teusch@hhu.de

[2] Department of Pharmacognosy, Faculty of Pharmacy, Ain Shams University, Cairo 11566, Egypt; mohamed.s.elnaggar@pharma.asu.edu.eg; aelissawy@pharma.asu.edu.eg; nehal.sabry@pharma.asu.edu.eg

[3] Centre of Drug Discovery Research and Development, Ain Shams University, Cairo 11566, Egypt

[4] Department of Chemistry, Faculty of Science, University of Dschang, P. O. Box 67, Dschang, Cameroon; remyteponno@gmail.com

[5] Laboratoire de Valorisation et Conservation des Ecosystèmes Arides (LVCEA), Faculté des Sciences de la Nature et de la Vie et Sciences de la Terre, Université de Ghardaia, B.P. 455, Ghardaïa, Algeria; bouras.noureddine@univ-ghardaia.dz

[6] Laboratoire de Biologie des Systèmes Microbiens (LBSM), Ecole Normale Supérieure de Kouba, Algiers, 16308, Algeria

[7] Department of Pharmacognosy and Medicinal Plants, Faculty of Pharmacy, Al-Azhar University, 11884 Cairo, Egypt; wael.afify@su.edu.eg

[8] Department of Pharmacognosy, Faculty of Pharmacy, Sinai University-Kantara Branch, 41636 Ismailia, Egypt.

* Correspondence: sherif_elsayed@pharma.asu.edu.eg (S.S.E.); abdelnassersingab@pharma.asu.edu.eg (A.N.B.S.); Nicole.Teusch@hhu.de (N.T.); Tel.: +49-211-81-14163; Fax +49-211-811-1923

Contents of Supporting Information

#	Contents	Page
1	Figure S1. HR-(+)ESI-MS spectrum of **1**.	S3
2	Figure S2. ^{1}H NMR spectrum of **1** in acetone-d_6 at 400 MHz.	S3
3	Figure S3. DEPTQ spectrum of **1** in acetone-d_6 at 100 MHz.	S4
4	Figure S4.^{1}H–^{1}H COSY spectrum of **1** in acetone-d_6 at 400 MHz.	S4
5	Figure S5. HMBC spectrum of **1** in acetone-d_6 at 400 MHz.	S5
6	Figure S6. HSQC spectrum of **1** in acetone-d_6 at 400 MHz.	S5
7	Figure S7. HR-(+)ESI-MS spectrum of **2**.	S6
8	Figure S8. ^{1}H NMR spectrum of **2** in DMSO-d_6 at 400 MHz.	S6
9	Figure S9. DEPTQ spectrum of **2** in DMSO-d_6 at 100 MHz.	S7
10	Figure S10. ^{1}H-^{1}H COSY spectrum of **2** in DMSO-d_6 at 400 MHz.	S7
11	Figure S11. HMBC spectrum of **2** in DMSO-d_6 at 400 MHz.	S8
12	Figure S12. HSQC spectrum of **2** in DMSO-d_6 at 400 MHz.	S8
13	Figure S13. NOESY spectrum of **2** in DMSO-d_6 at 400 MHz.	S9
14	Figure S14. LR-ESI-MS spectrum of **3**.	S9
15	Figure S15. ^{1}H NMR spectrum of **3** in DMSO-d_6 at 400 MHz.	S10
16	Figure S16. DEPTQ spectrum of **3** in DMSO-d_6 at 100 MHz.	S10
17	Figure S17. ^{1}H-^{1}H COSY spectrum of **3** in DMSO-d_6 at 400 MHz.	S11
18	Figure S18. HMBC spectrum of **3** in DMSO-d_6 at 400 MHz.	S11
19	Figure S19. HSQC spectrum of **3** in DMSO-d_6 at 400 MHz.	S12
20	Figure S20. NOESY spectrum of **3** in DMSO-d_6 at 400 MHz.	S12
21	Figure S21. LR-ESI-MS spectrum of **4**.	S13
22	Figure S22. ^{1}H NMR spectrum of **4** in DMSO-d_6 at 400 MHz	S13
23	Figure S23. DEPTQ spectrum of **4** in DMSO-d_6 at 100 MHz.	S14
24	Figure S24. ^{1}H-^{1}H COSY spectrum of **4** in DMSO-d_6 at 400 MHz.	S14
25	Figure S25. HMBC spectrum of **4** in DMSO-d_6 at 400 MHz.	S15
26	Figure S26. HSQC spectrum of **4** in DMSO-d_6 at 400 MHz.	S15
27	Figure S27. NOESY spectrum of **4** in DMSO-d_6 at 400 MHz.	S16
28	Figure S28. LR-ESI-MS spectrum of **5**.	S16
29	Figure S29. ^{1}H NMR spectrum of **5** in $CDCl_3$-*d* at 400 MHz	S17
30	Figure S30. DEPTQ spectrum of **5** in $CDCl_3$-*d* at 100 MHz.	S17
31	Figure S31. ^{1}H-^{1}H COSY spectrum of **5** in $CDCl_3$-*d* at 400 MHz.	S18
32	Figure S32. HMBC spectrum of **5** in $CDCl_3$-*d* at 400 MHz.	S18
33	Figure S33. HSQC spectrum of **5** in $CDCl_3$-*d* at 400 MHz.	S19
34	Figure S34. ITS sequence for the fungal strain MNP-GP4	S19

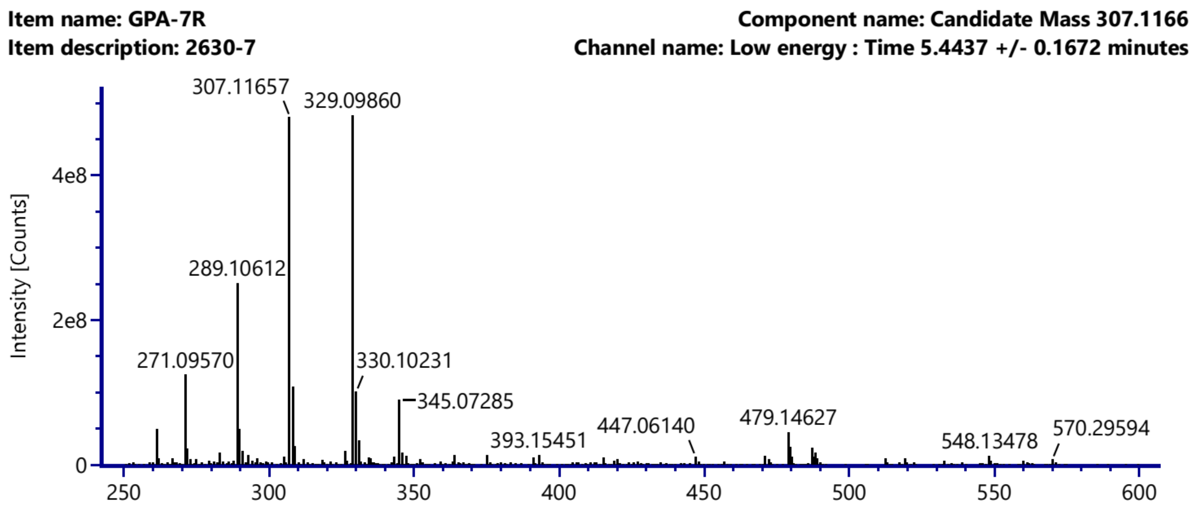

Figure S1. HR-(+)ESI-MS spectrum of **1**.

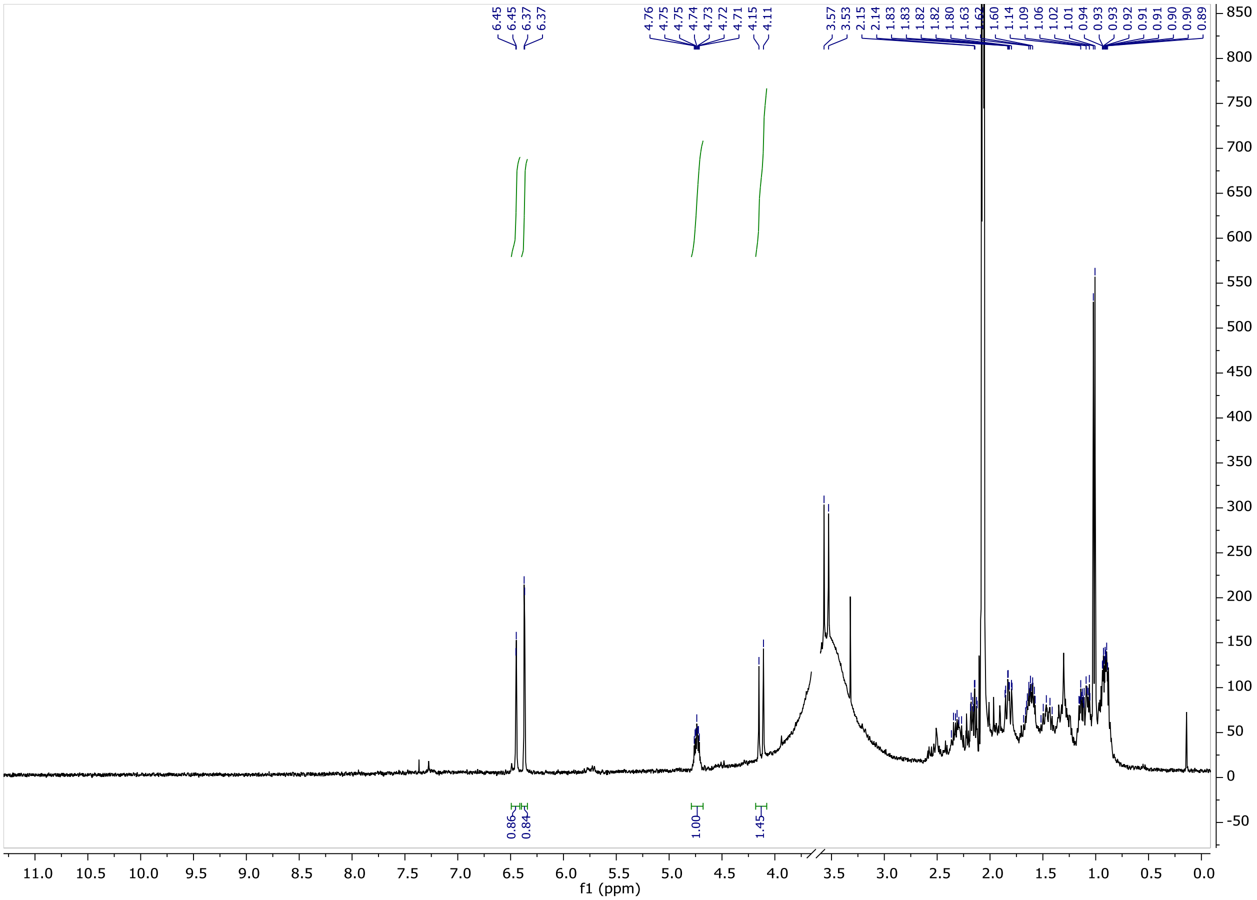

Figure S2. ^{1}H NMR spectrum of **1** in acetone-d_6 at 400 MHz.

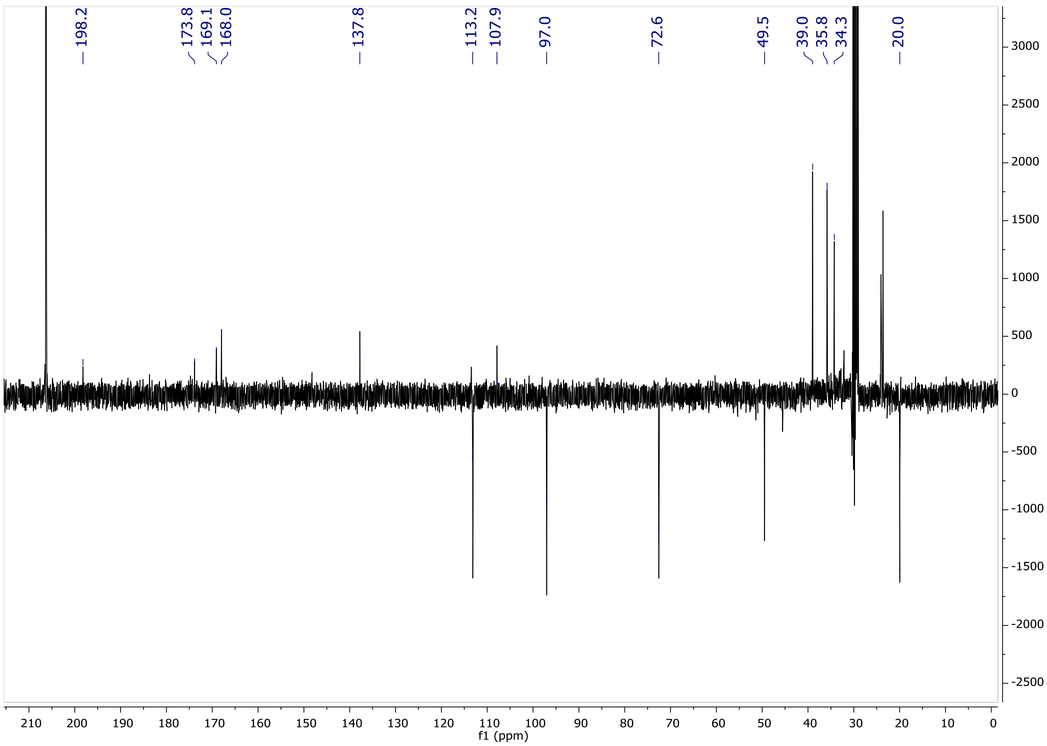

Figure S3. DEPTQ spectrum of **1** in acetone-*d*6 at 100 MHz.

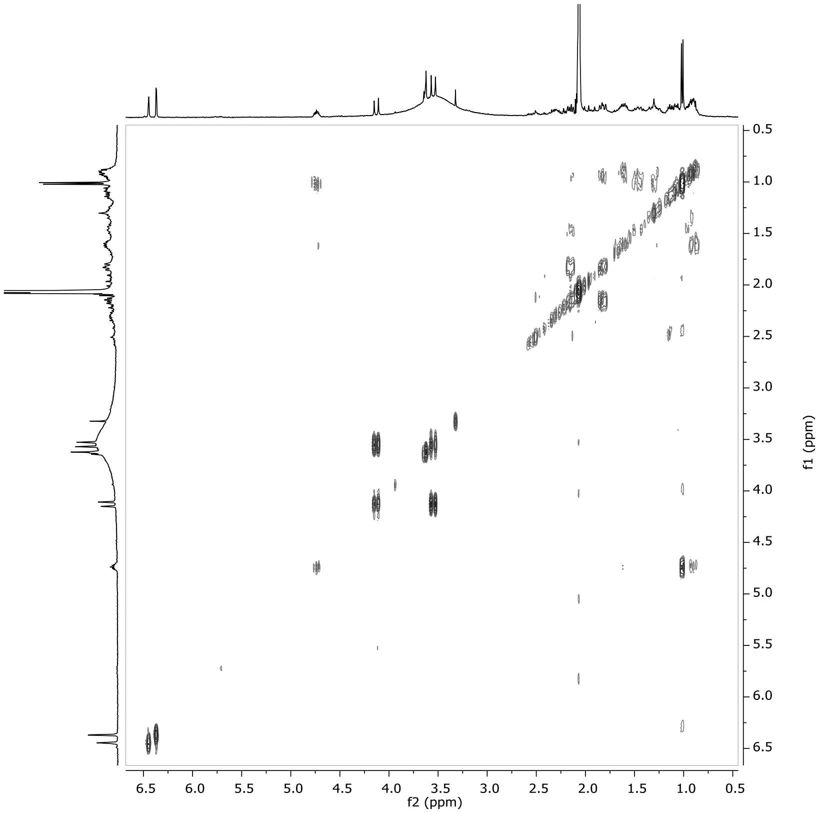

Figure S4. ^{1}H–^{1}H COSY spectrum of **1** in acetone-*d*6 at 400 MHz.

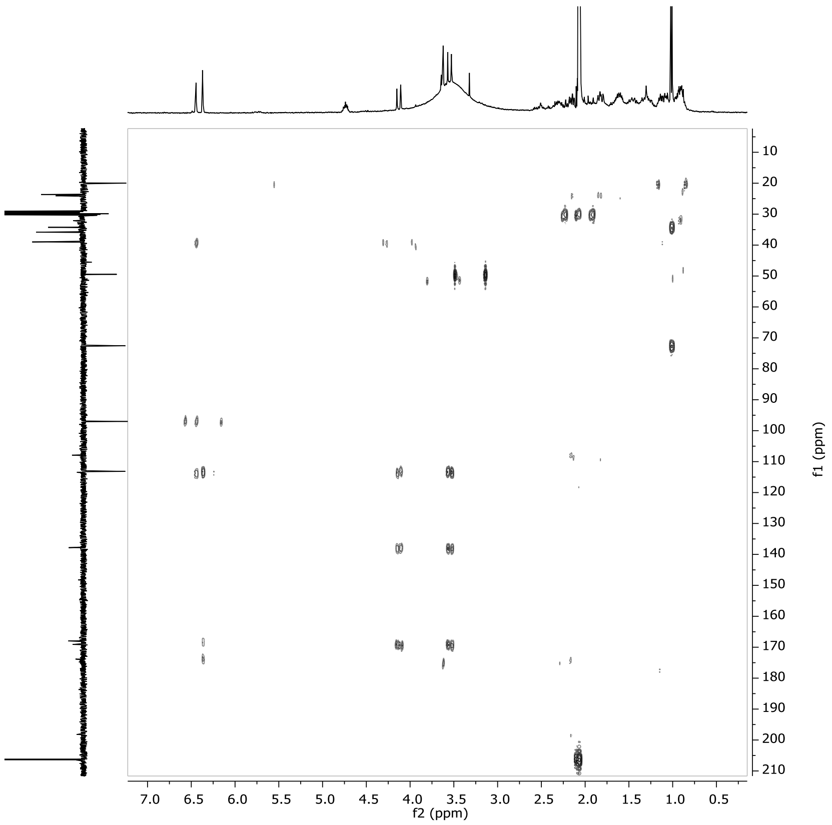

Figure S5. HMBC spectrum of **1** in acetone-d_6 at 400 MHz.

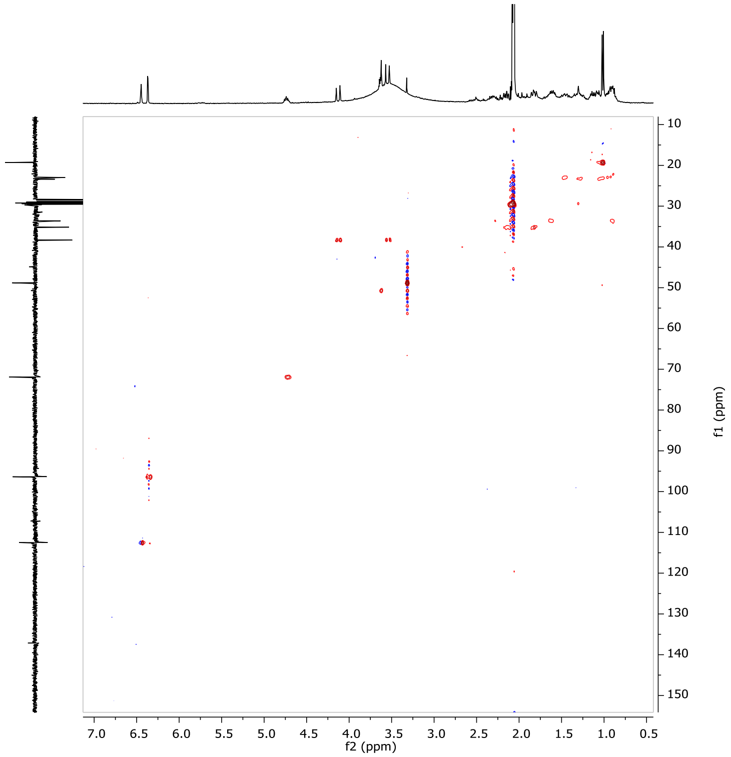

Figure S6. HSQC spectrum of **1** in acetone-d_6 at 400 MHz.

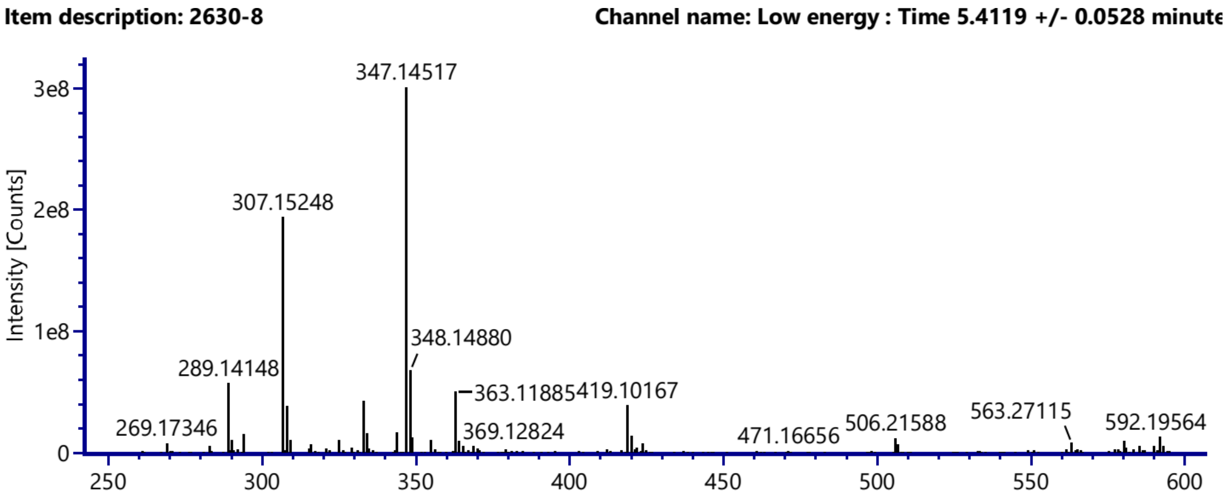

Figure S7. HR-(+)ESI-MS spectrum of **2**.

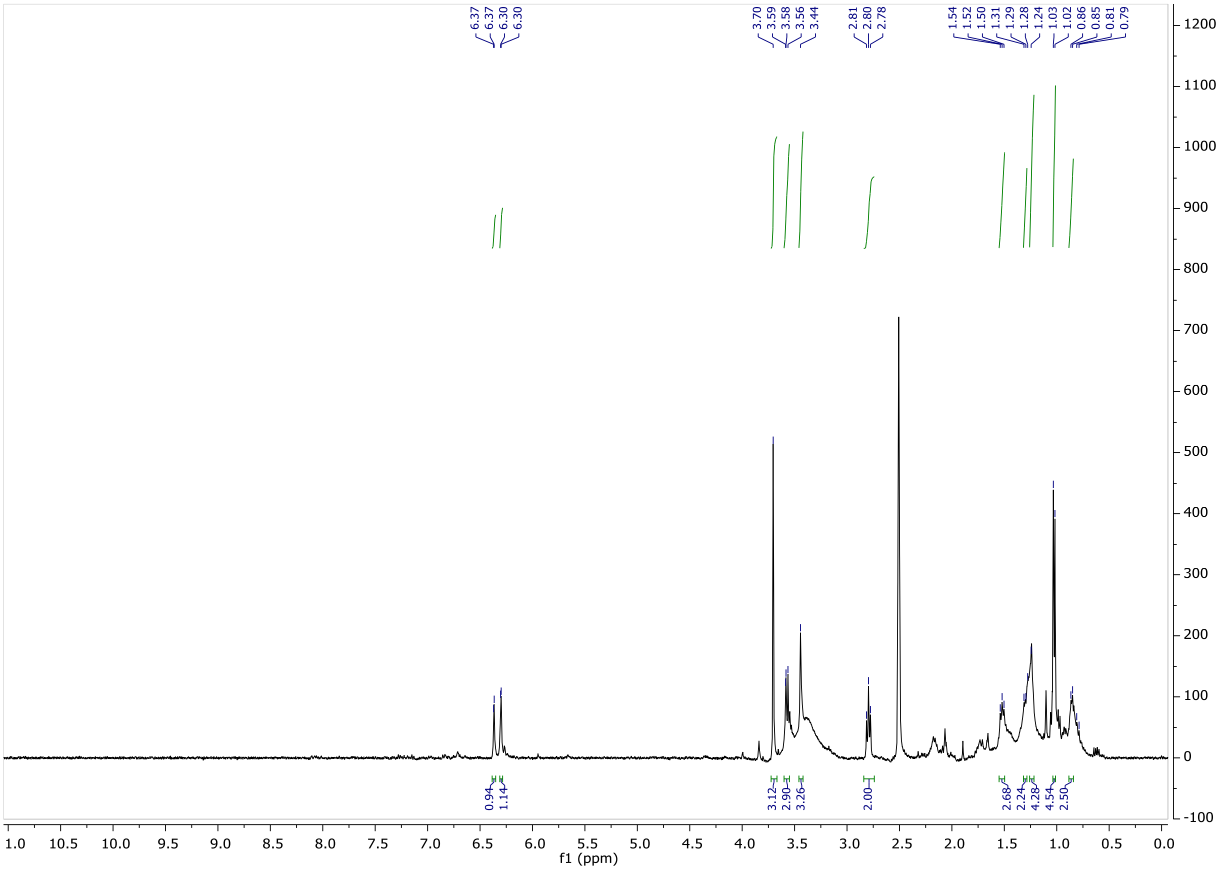

Figure S8. ^{1}H NMR spectrum of **2** in DMSO-d_6 at 400 MHz.

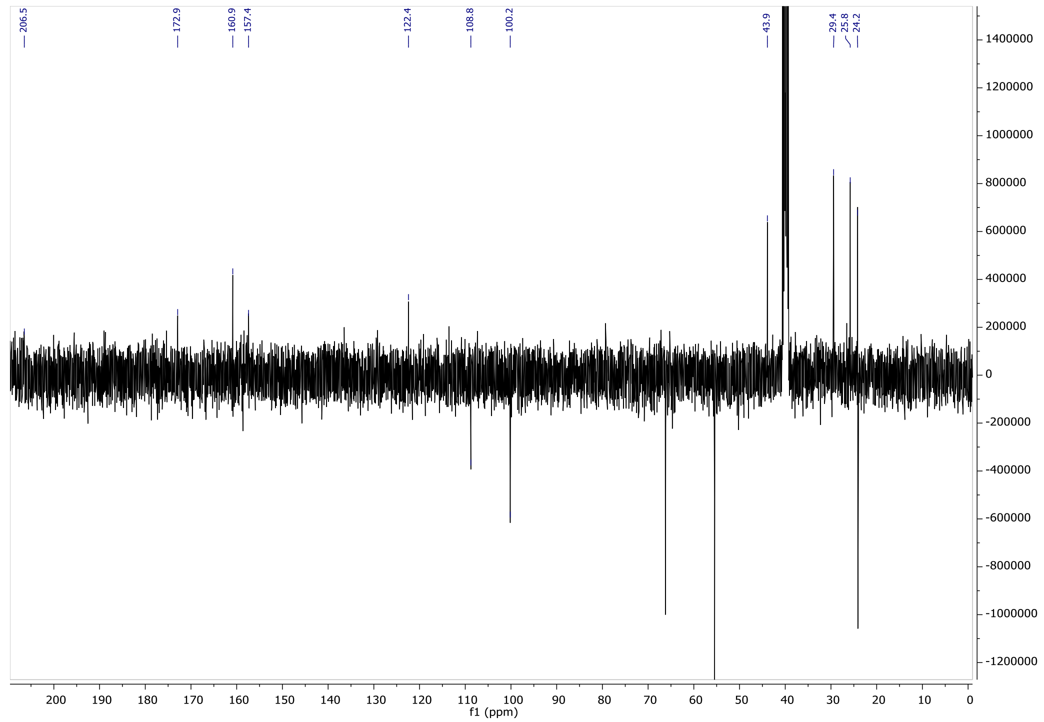

Figure S9. ^{1}H NMR spectrum of **2** in DMSO-d_6 at 100 MHz.

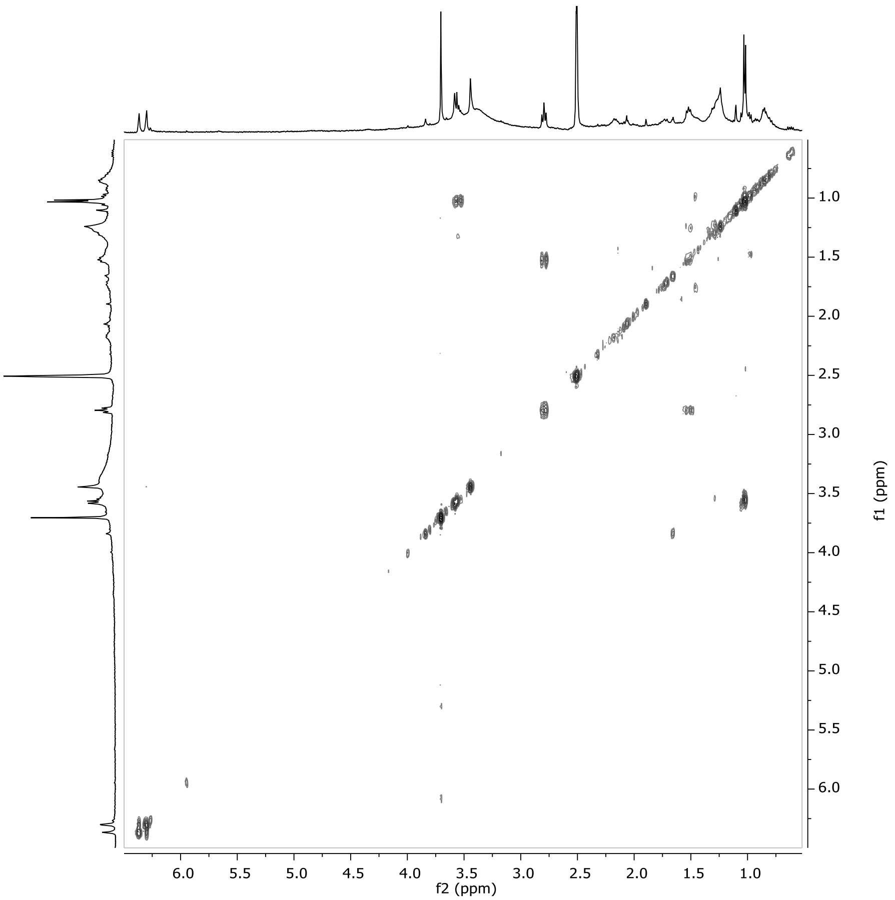

Figure S10. ^{1}H-^{1}H COSY spectrum of **2** in DMSO-d_6 at 400 MHz.

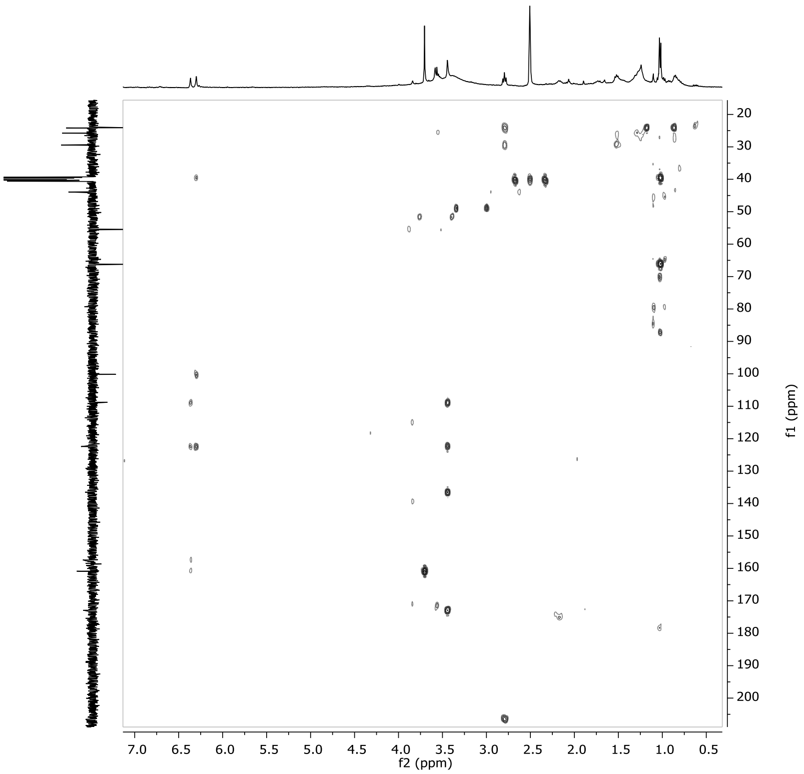

Figure S11. HMBC spectrum of **2** in DMSO-d_6 at 400 MHz.

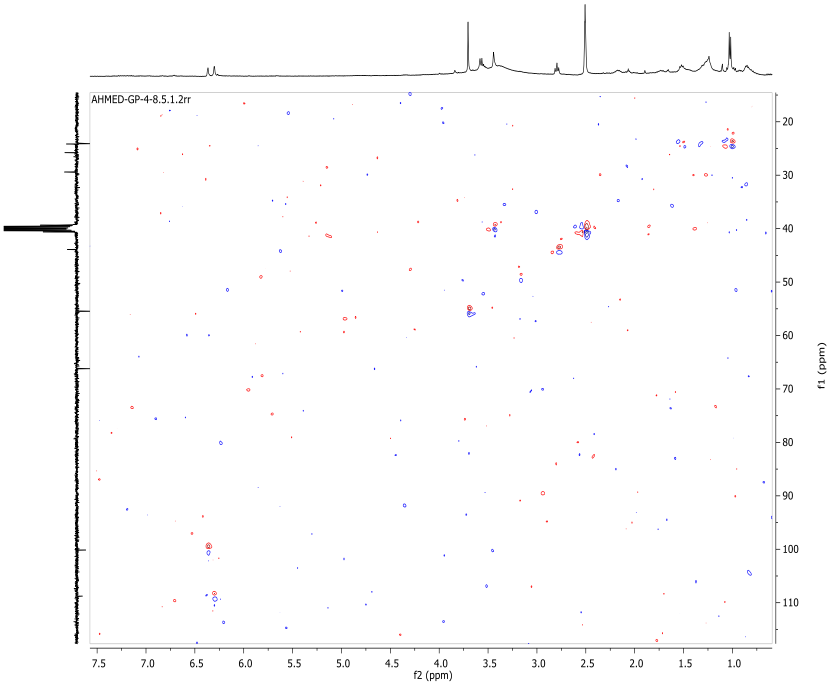

Figure S12. HSQC spectrum of **2** in DMSO-d_6 at 400 MHz.

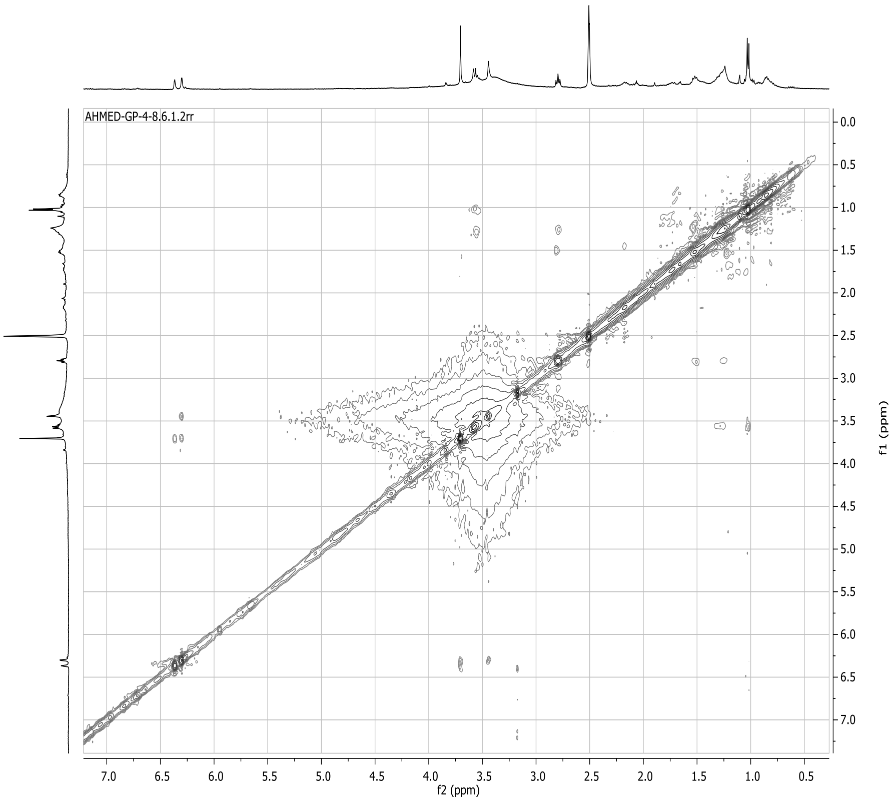

Figure S13. NOESY spectrum of **2** in DMSO-d_6 at 400 MHz.

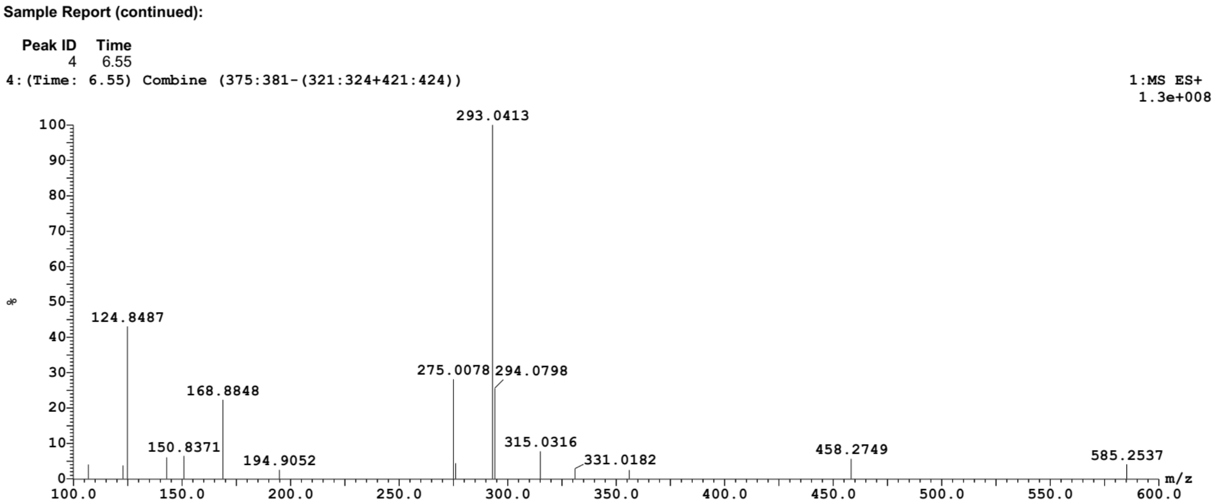

Figure S14. LR-ESI-MS spectrum of **3**.

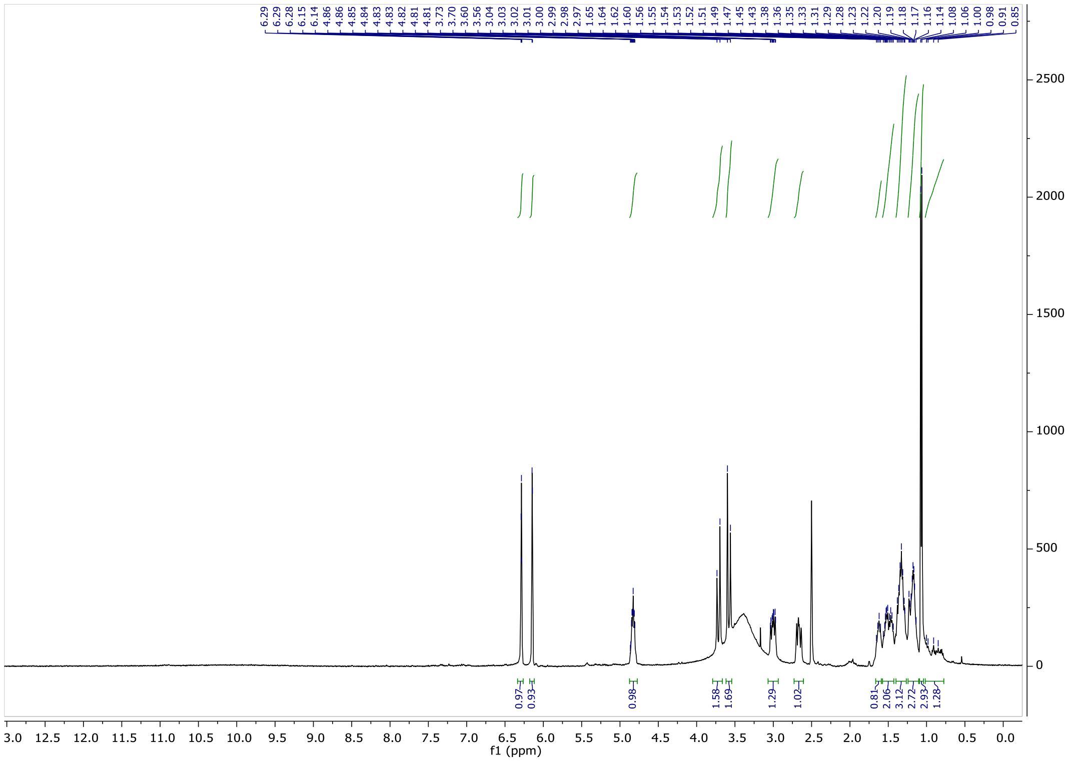

Figure S15. ^{1}H NMR spectrum of **3** in DMSO-d_6 at 400 MHz.

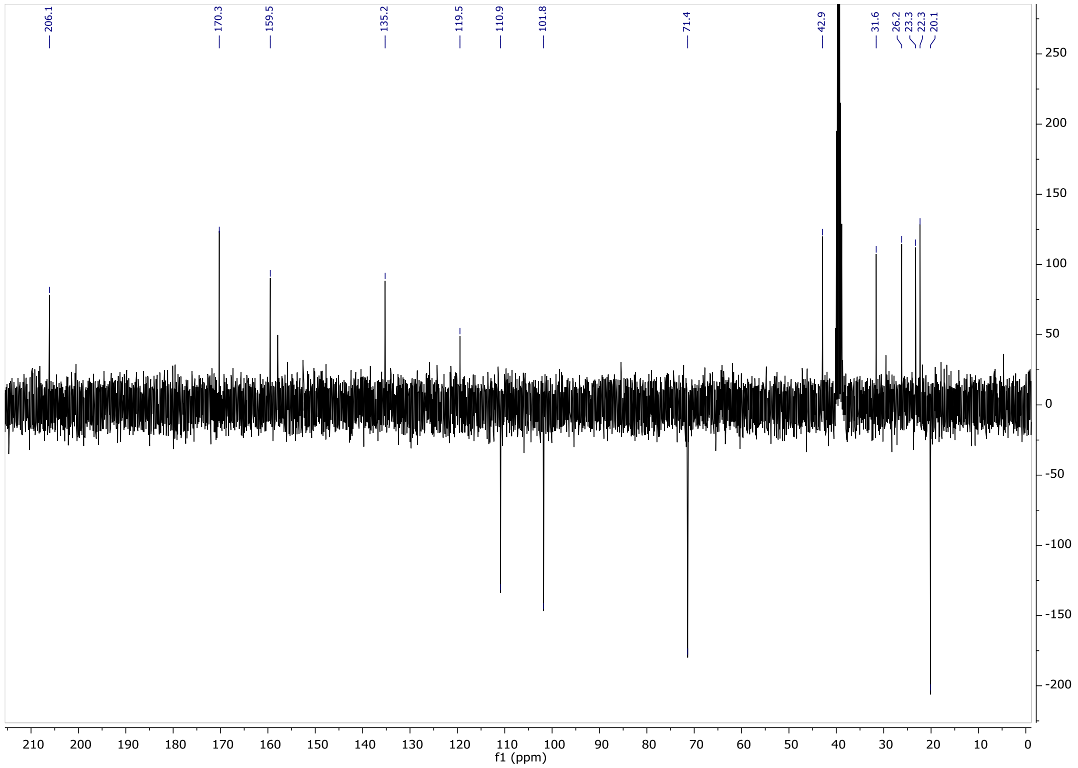

Figure S16. DEPTQ spectrum of **3** in DMSO-d_6 at 100 MHz.

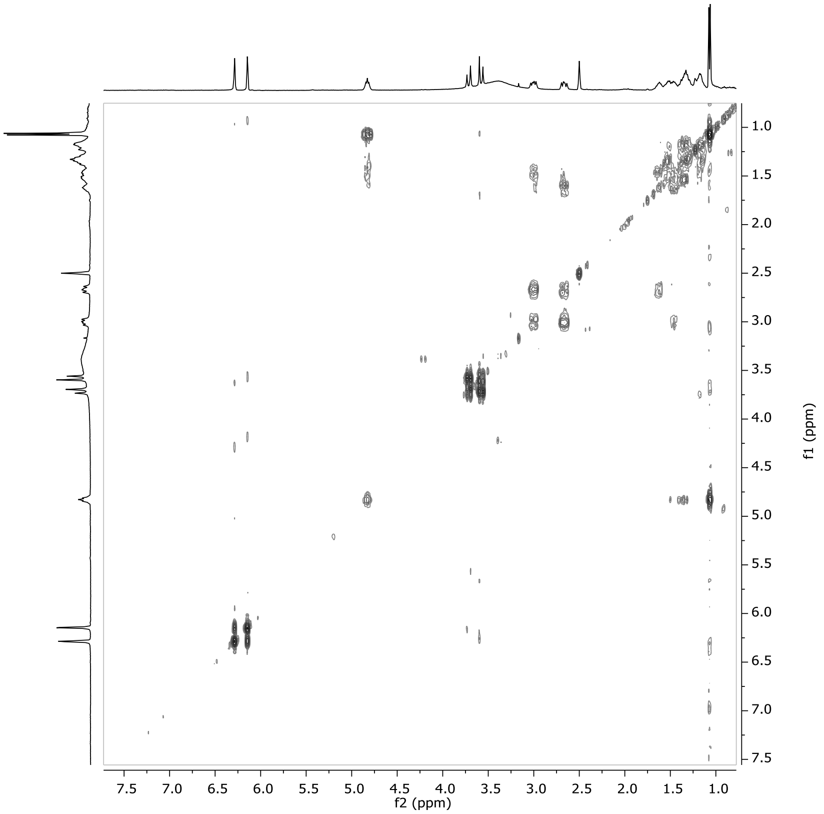

Figure S17. ^{1}H-^{1}H COSY spectrum of **3** in DMSO-d_6 at 400 MHz.

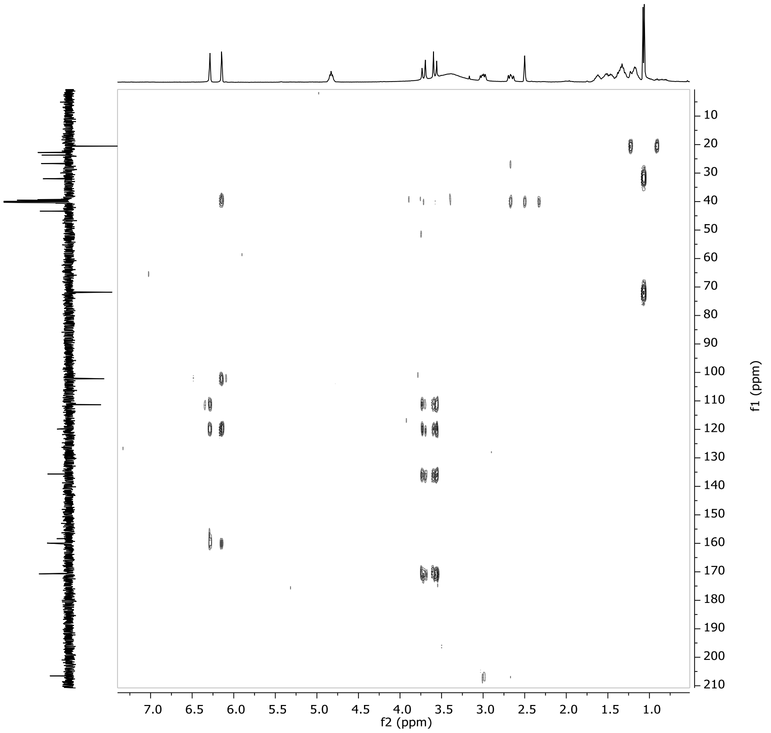

Figure S18. HMBC spectrum of **3** in DMSO-d_6 at 400 MHz.

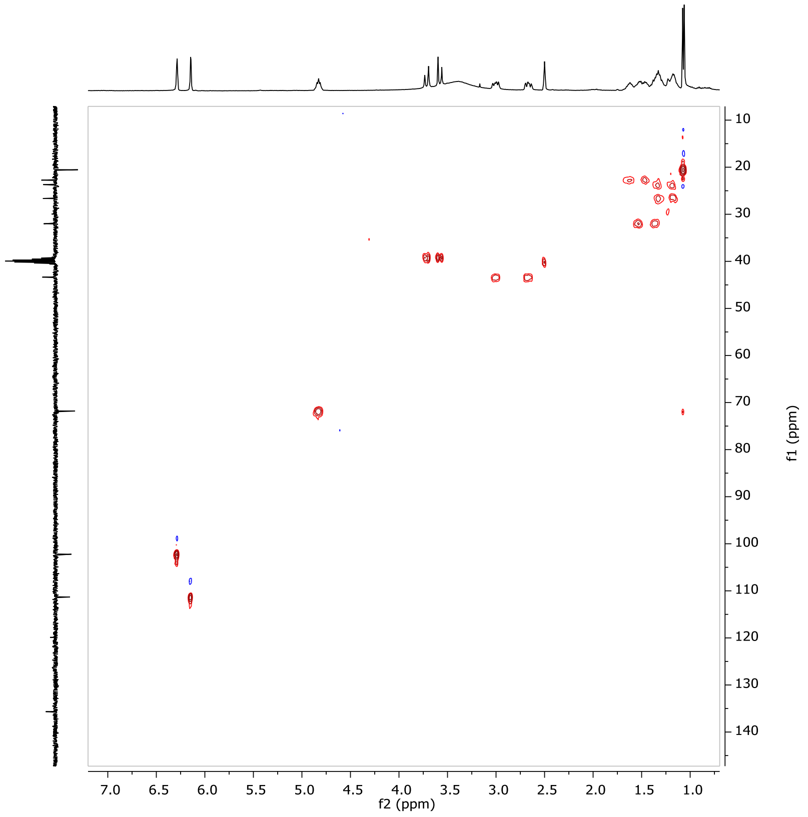

Figure S19. HSQC spectrum of **3** in DMSO-d_6 at 400 MHz.

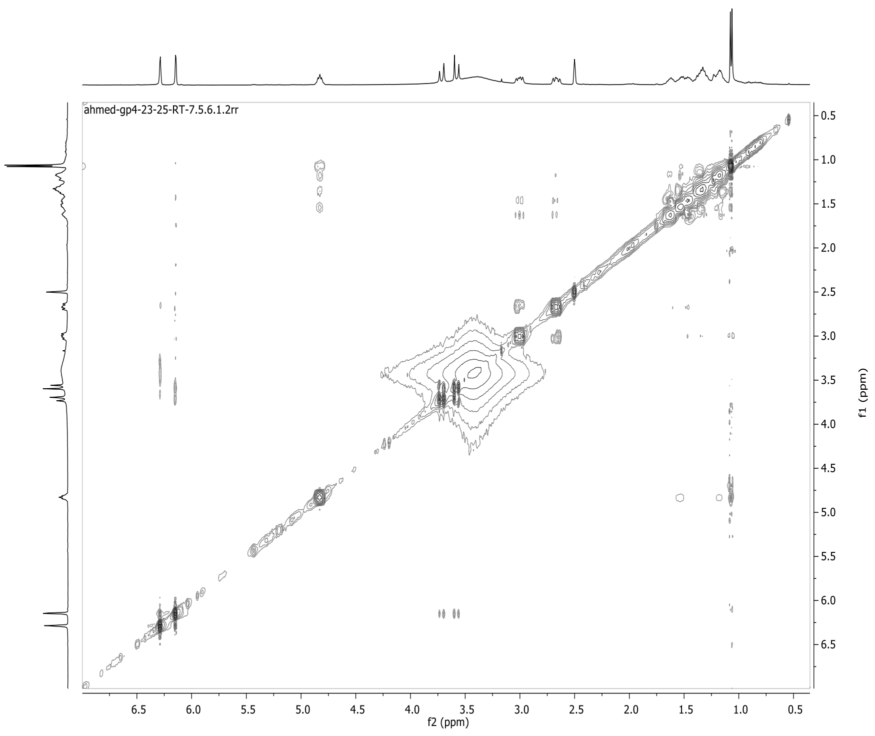

Figure S20. NOESY spectrum of **3** in DMSO-d_6 at 400 MHz.

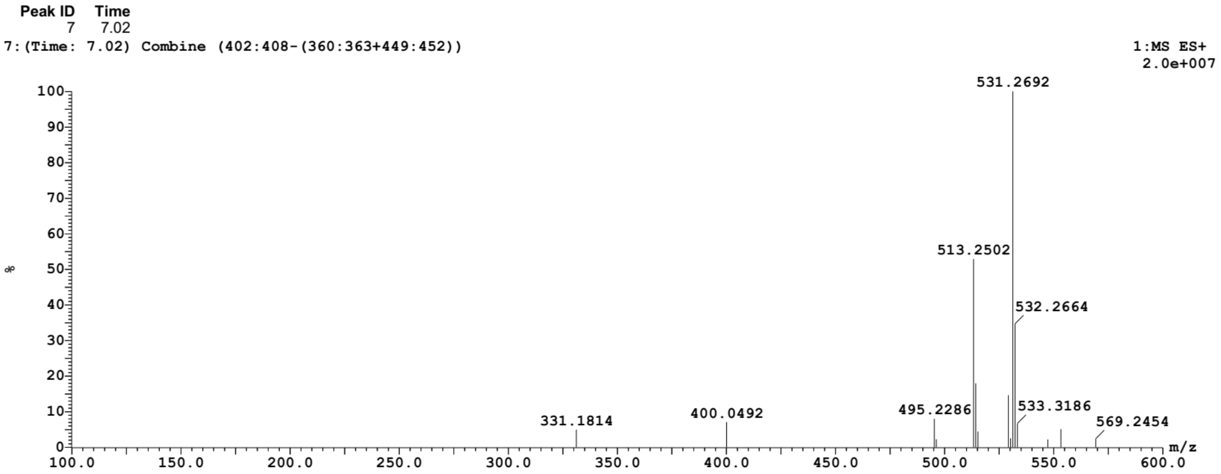

Figure S21. LR-(+)ESI-MS spectrum of **4**.

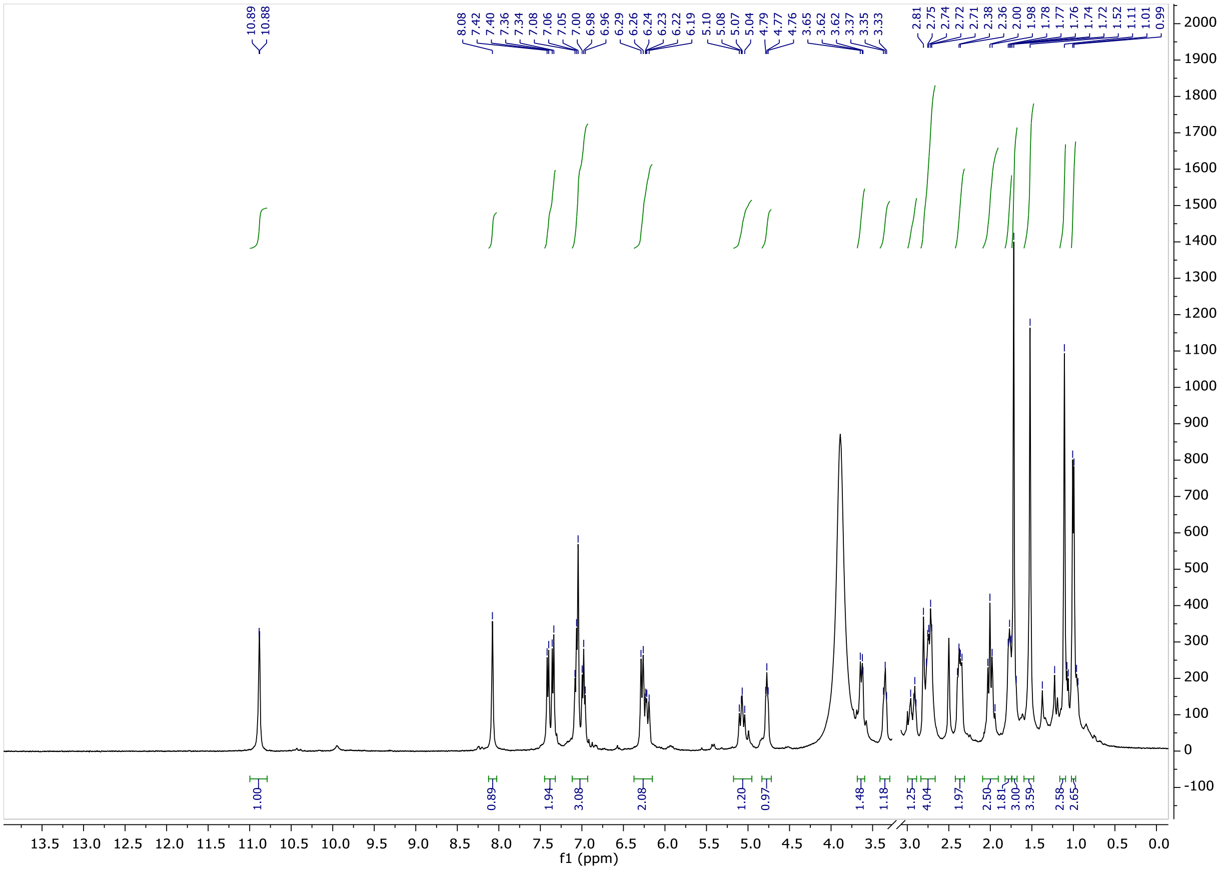

Figure S22. ^{1}H NMR spectrum of **4** in DMSO-d_6 at 400 MHz.

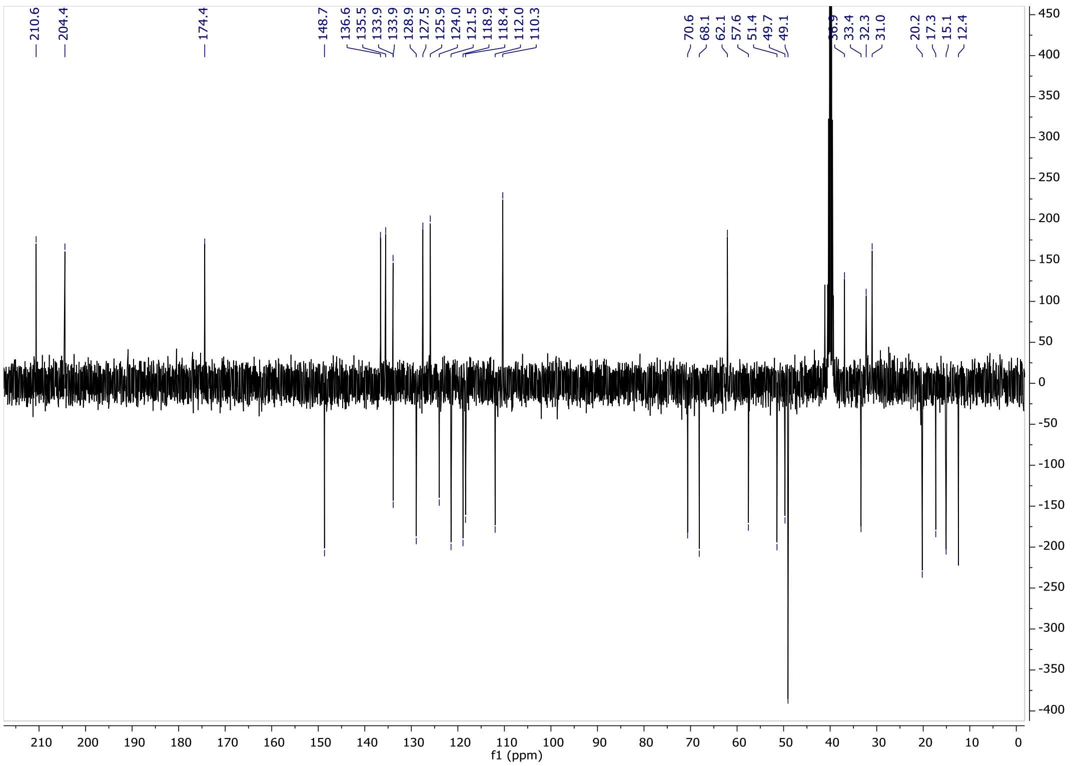

Figure S23. DEPTQ spectrum of **4** in DMSO-d_6 at 100 MHz.

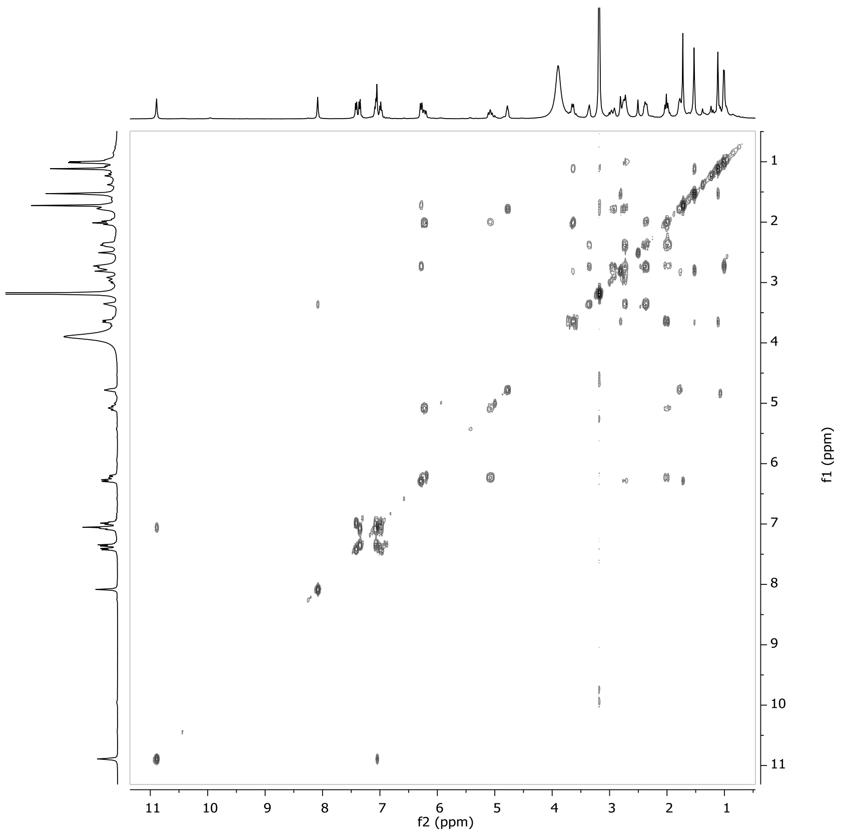

Figure S24. ^{1}H-^{1}H COSY spectrum of **4** in DMSO-d_6 at 400 MHz.

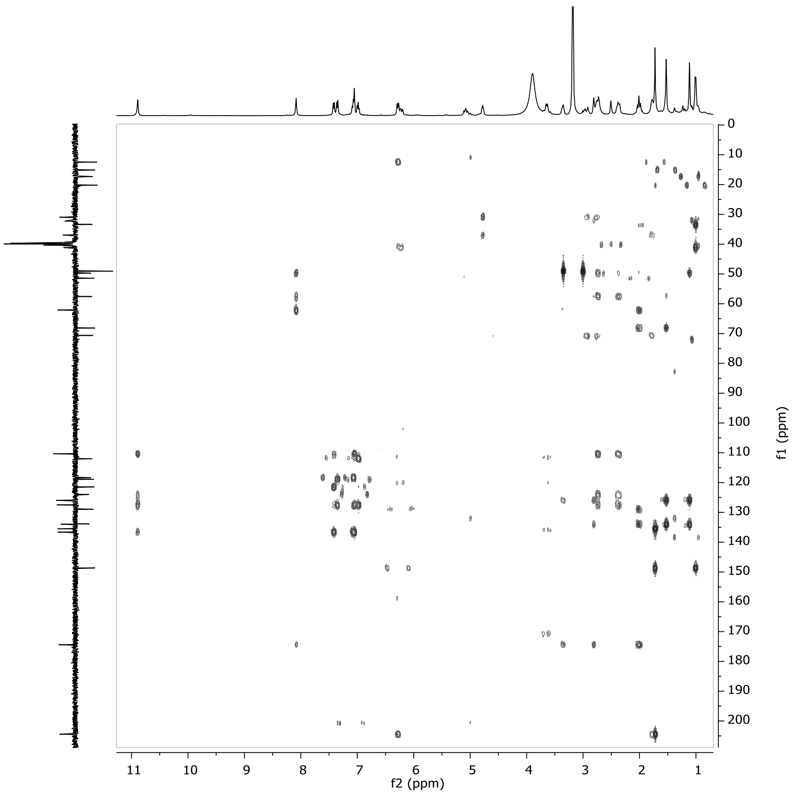

Figure S25. HMBC spectrum of **4** in DMSO-d_6 at 400 MHz.

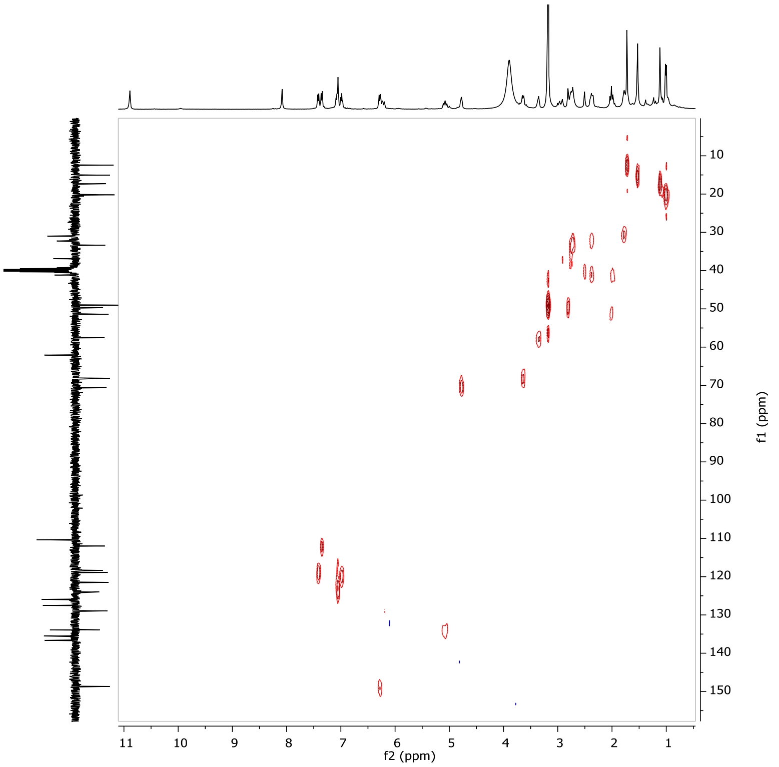

Figure S26. HSQC spectrum of **4** in DMSO-d_6 at 400 MHz.

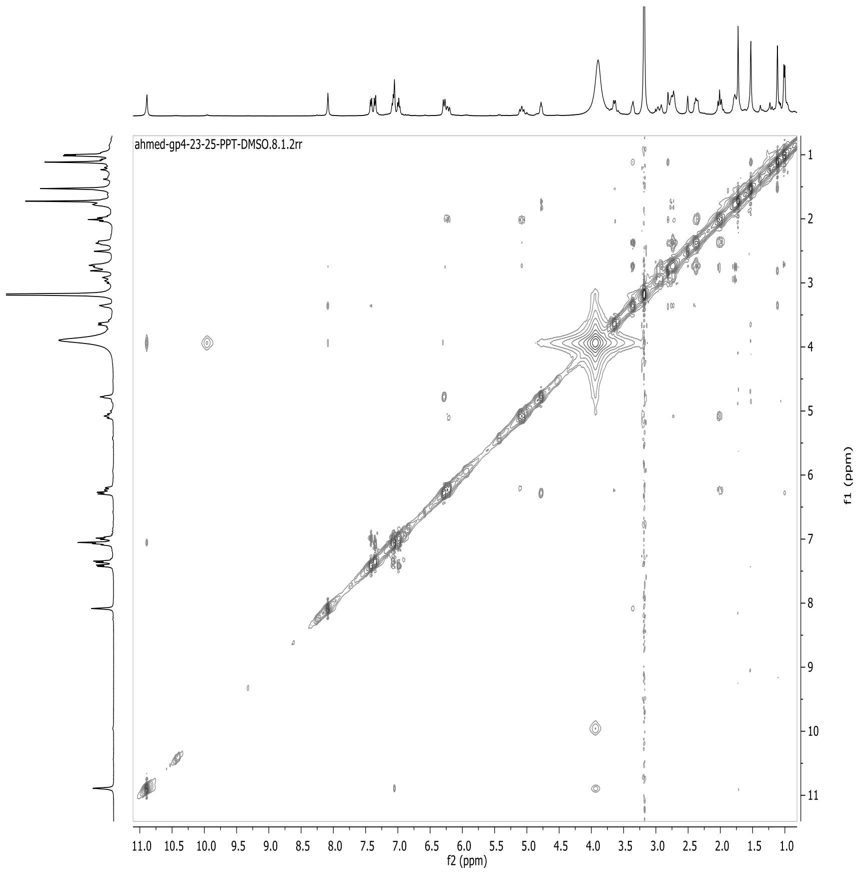

Figure S27. NOESY spectrum of **4** in DMSO-d_6 at 400 MHz.

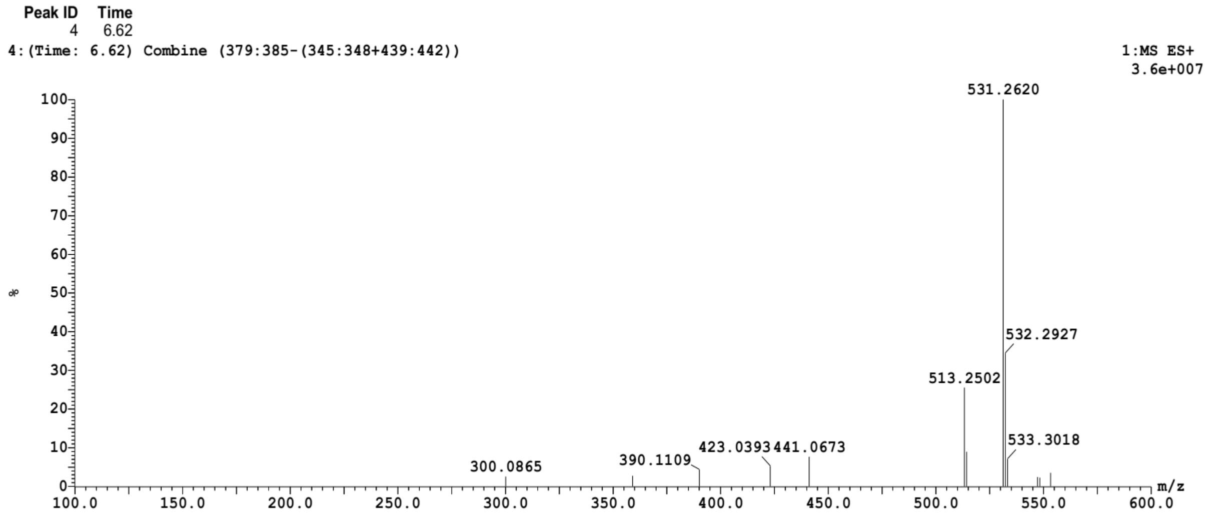

Figure S28. Positive ESIMS spectrum of compound **5**.

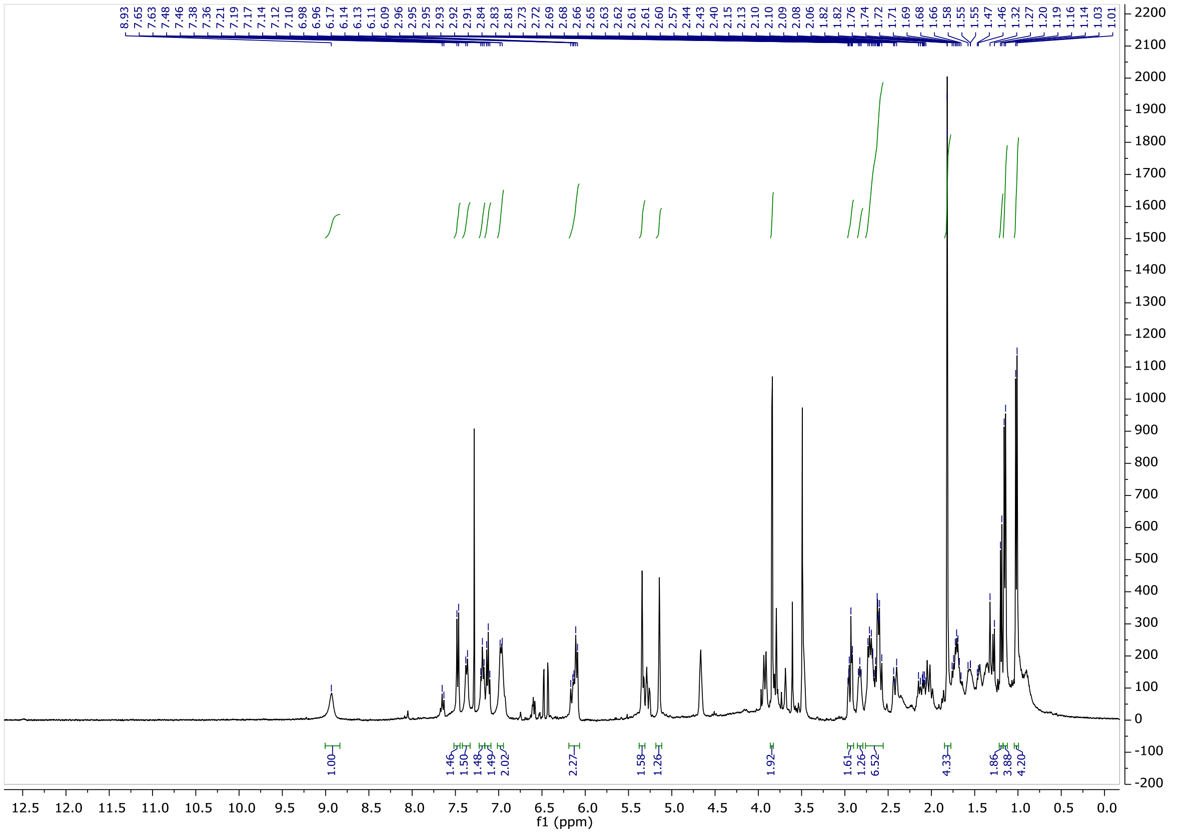

Figure S29. ^{1}H NMR spectrum of **5 in** chloroform-*d* at 400 MHz.

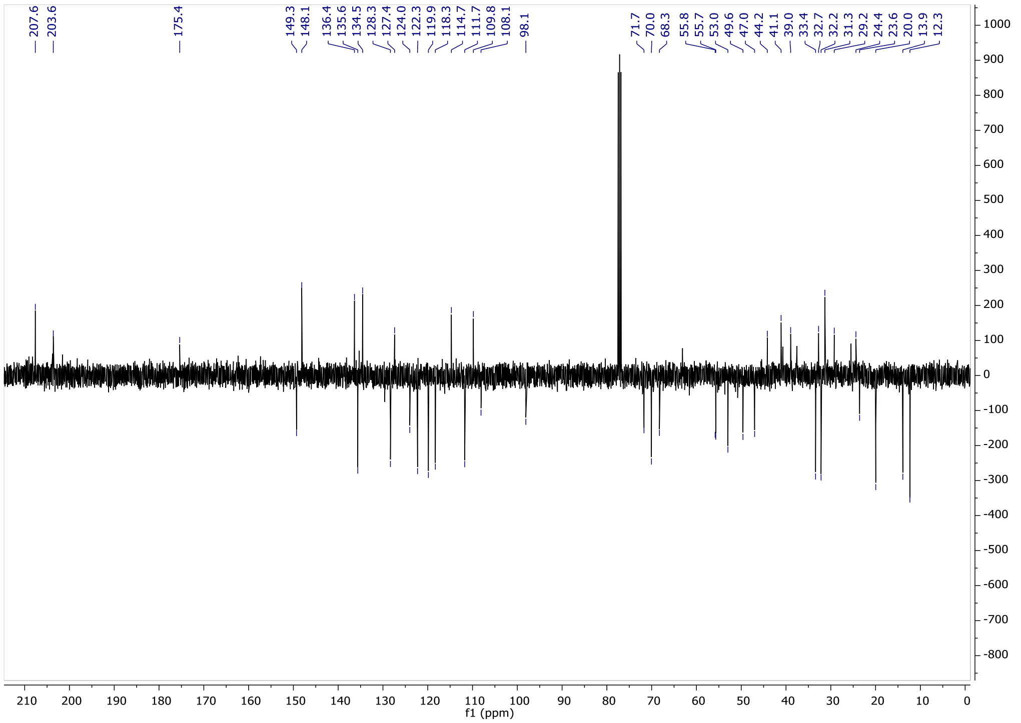

Figure S30. DEPTQ spectrum of **5** in chloroform-*d* at 100 MHz.

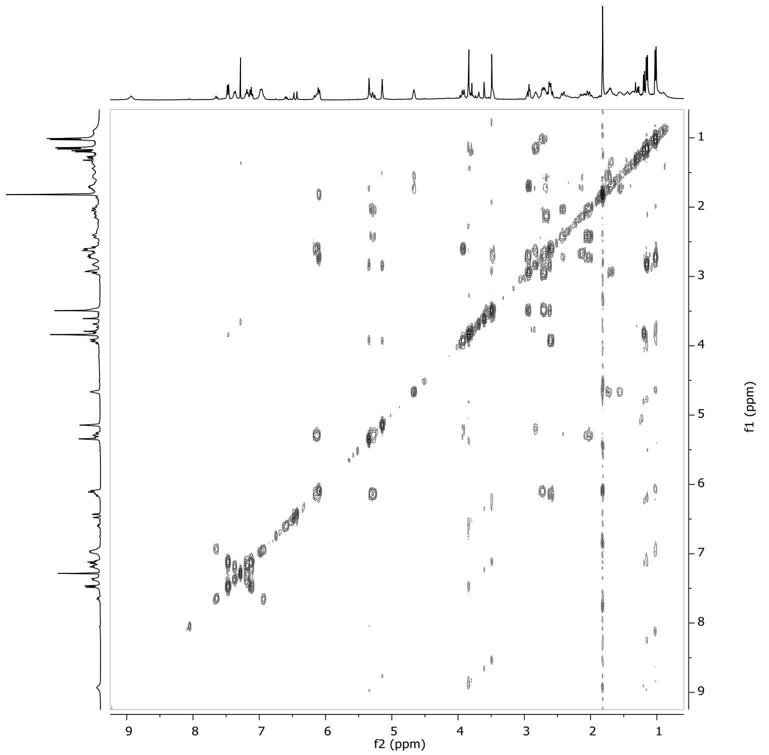

Figure S31. ^{1}H-^{1}H COSY spectrum of **5** in chloroform-*d* at 400 MHz.

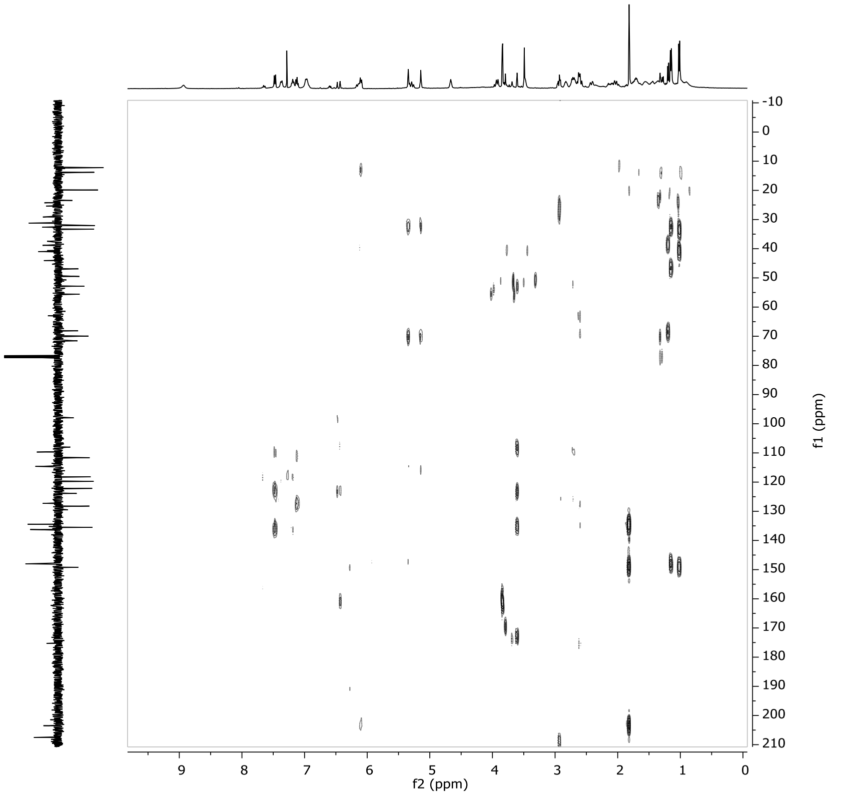

Figure S32. HMBC spectrum of **5** in chloroform-*d* at 400 MHz.

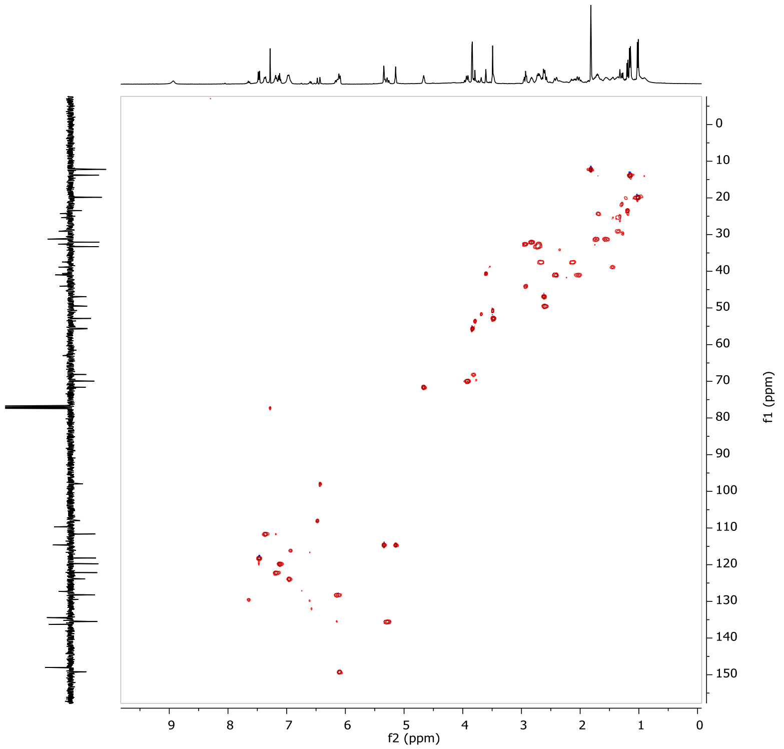

Figure S33. HSQC spectrum of **5** in chloroform-*d* at 400 MHz.

GGGCCCGCCTCCACGGCCGCCGGGGGGCTCCTGCCCCCGGGCCCGCGCCCGCCGAAGCCCCCCCTTGAACGCTGTCTGAAGTTTGCAGTCTG
AGAAACTAGCTAAATTAGTTAAAACTTTCAACAACGGATCTCTTGGTTCCGGCATCGATGAAGAACGCAGCGAAATGCGATAACTAATGTGAA
TTGCAGAATTCAGTGAATCATCGAGTCTTTGAACGCACATTGCGCCCTCTGGTATTCCGGAGGGCATGCCTGTCCGAGCGTCATTGCTGCCCTCA
AGCACGGCTTGTGTGTTGGGCCCCCGTCCCCCCCTCTGCCGGGGGGACGGGCCCGAAAGGCAGCGGCGGCACCGCGTCCGGTCCTCGAGCGT
ATGGGGCTTCGTCACCCGCTCTTGTAGGCCCGGCCGGCGCCAGCCGACCCCAACCCTAAATTTTTTTC

Figure S34. ITS sesquence for the fungal strain MNP-GP4
